# Higher habitual intakes of flavonoids and flavonoid-rich foods are associated with a lower incidence of type 2 diabetes in the UK Biobank cohort

**DOI:** 10.1038/s41387-024-00288-0

**Published:** 2024-05-22

**Authors:** Alysha S. Thompson, Amy Jennings, Nicola P. Bondonno, Anna Tresserra-Rimbau, Benjamin H. Parmenter, Claire Hill, Aurora Perez-Cornago, Tilman Kühn, Aedín Cassidy

**Affiliations:** 1 The Institute for Global Food Security, School of Biological Sciences, Queen’s University Belfast, Belfast, Northern Ireland UK; 2 Danish Cancer Society Research Centre (DCRC), Copenhagen, Denmark; 3 Nutrition & Health Innovation Research Institute, School of Medical and Health Sciences, Edith Cowan University, Joondalup, WA Australia; 4 Department of Nutrition, Food Science and Gastronomy, XIA, School of Pharmacy and Food Sciences, INSA, University of Barcelona, 08921 Barcelona, Spain; 5 Centro de Investigación Biomédica en Red Fisiopatología de la Obesidad y la Nutrición (CIBEROBN), Institute of Health Carlos III, 28029 Madrid, Spain; 6 Centre for Public Health, Queen’s University Belfast, Belfast, UK; 7 Nuffield Department of Population Health, Cancer Epidemiology Unit, University of Oxford, Oxford, UK; 8 Department of Nutritional Sciences, University of Vienna, Vienna, Austria; 9 Medical University of Vienna, Center for Public Health, Vienna, Austria; 10 Heidelberg Institute of Global Health (HIGH), Faculty of Medicine and University Hospital, Heidelberg, Germany

**Keywords:** Type 2 diabetes, Risk factors, Epidemiology, Lifestyle modification

## Abstract

**Aim:**

To examine the associations of a diet high in flavonoid-rich foods, as reflected by a “Flavodiet Score” (FDS), the major individual food contributors to flavonoid intake, and flavonoid subclasses with type 2 diabetes (T2D) risk in the UK Biobank cohort.

**Materials and methods:**

Flavonoid intakes were estimated from ≥2 dietary assessments among 113,097 study participants [age at enrolment: 56 ± 8 years; 57% female] using the U.S Department of Agriculture (USDA) databases. Multivariable Cox proportional hazards models were used to investigate associations between dietary exposures and T2D.

**Results:**

During 12 years of follow-up, 2628 incident cases of T2D were identified. A higher FDS (compared to lower [Q4 vs. Q1]), characterised by an average of 6 servings of flavonoid-rich foods per day, was associated with a 26% lower T2D risk [HR: 0.74 (95% CI: 0.66–0.84), p_trend_ = <0.001]. Mediation analyses showed that lower body fatness and basal inflammation, as well as better kidney and liver function partially explain this association. In food-based analyses, higher intakes of black or green tea, berries, and apples were significantly associated with 21%, 15%, and 12% lower T2D risk. Among individual flavonoid subclasses, 19–28% lower risks of T2D were observed among those with the highest, compared to lowest intakes.

**Conclusions:**

A higher consumption of flavonoid-rich foods was associated with lower T2D risk, potentially mediated by benefits to obesity/sugar metabolism, inflammation, kidney and liver function. Achievable increases in intakes of specific flavonoid-rich foods have the potential to reduce T2D risk.

## Introduction

Type 2 diabetes (T2D) remains one of the top four major non-communicable diseases (NCDs), contributing to >4 million diabetes-related deaths annually [1]. Across the globe, 415 million people live with diabetes [1], with an estimated 90% of these people having T2D. Widely preventable, T2D is associated with higher risk of cardiovascular disease, dementia, and cancer, causing extensive global economic burden in healthcare costs [2]. Modifiable risk factors such as overweight and obesity are considered as major contributors to T2D, and thus diet is crucial for primary prevention [3, 4]. Previous studies suggest a preventive association of healthy plant-based dietary patterns with T2D risk beyond their effects on energy balance [5].

Favourable health effects of plant-food consumption have been attributed to flavonoids, a class of polyphenolic compounds that are commonly found in fruits, vegetables, dark chocolate, tea, and red wine [6]. Flavonoids have been categorised into six main subclasses (flavanones, flavones, flavan-3-ols (including proanthocyanidins), flavonols, anthocyanins, and isoflavones), with different bioavailability and bioactivity [7, 8]. Several prospective studies have reported inverse associations between numerous flavonoid subclasses and incident T2D risk [9]. There is growing evidence from short-term randomised controlled trials (RCTs) to suggest that higher intakes of various flavonoid subclasses, namely anthocyanins and flavan-3-ols consumed through flavonoid-rich food sources, such as cocoa, tea and blueberries improves insulin sensitivity, insulin resistance, and lipid profiles [10–12].

To better understand the role of dietary flavonoid intake in T2D prevention, and given that flavonoids are typically consumed as part of a dietary pattern through flavonoid-rich foods, we investigated whether a flavonoid-rich diet represented by a novel Flavodiet Score (FDS), which is associated with lower mortality risk [13], is associated with incident T2D in a large-scale population-based study, the UK Biobank, for the first time. We further carried out mediation analyses to evaluate which potential biological mechanisms may mediate associations between a flavonoid-rich diet and T2D risk. We also aimed to evaluate associations between the major food contributors to flavonoid intake and habitual intakes of flavonoid subclasses with incident T2D.

## Materials and methods

### Study population

The UK Biobank is a large-scale population-based prospective cohort study of >500,000 participants aged between 40 and 69 years and recruited between 2006 and 2010 from the UK. Participants attended one of 22 assessment centres located across England, Scotland, and Wales, where they completed a baseline assessment comprising a touchscreen questionnaire and a series of physical assessments. Further information on the study protocol has been previously reported [14]. All UK Biobank participants provided written informed consent at recruitment and the study received ethical approval from the NHS North West Multicentre Research Ethics Committee (Ref. 11/NW/0382).

Participants who completed fewer than two 24-h dietary assessments (*n* = 372,173), had implausible energy intakes (>17,573 KJ or <3347 KJ for men and >14,644 KJ or < 2092 KJ for women [15]) (*n* = 3953), or withdrew their consent during the study follow-up were excluded from the analysis. Participants were also excluded if they had diabetes (*n* = 5009), CVD (*n* = 1868), cancer (*n* = 5920), were diagnosed with T2D before the last 24-h dietary assessment (*n* = 301), or if they were pregnant at baseline assessment (*n* = 46) (Supplemental Fig. S1).

### Flavodiet Score (FDS)

Further details on the Oxford WebQ dietary assessment tool, including the method used to estimate flavonoid intakes are provided in the Supplementary Appendix. The top three foods that contributed the highest percent to total flavonoid and flavonoid subclass intakes were considered eligible for inclusion in the FDS (excluding fruit juices). In addition, dark chocolate was also considered eligible for inclusion within the score as it typically contains high concentrations of flavonoids. However, in this UK population, intakes of dark chocolate were low, with a median (range) of 0 (0–2.5) servings/day, and thus, did not contribute highly to flavonoid intakes. Based on mean intakes (servings/day) from a minimum of two 24-h dietary assessments, the score was made up of ten Oxford WebQ food items (tea (black and green), red wine, apples, berries, grapes, oranges (including satsumas), grapefruit, sweet peppers, onions, and dark chocolate). A final FDS was calculated by summing the total number of servings consumed across the ten selected food items. Tea (black and green) intakes were capped at a maximum of 4 servings/day due to the high intakes reported in this British population (median (range) 2.3 (0–11.5)). From this, the FDS was categorised into sex-specific quartiles. As we were interested in flavonoids rather than alcohol consumption, (a) we adjusted for total alcohol consumption in our statistical models on the FDS and T2D risk, and (b) we re-ran our statistical analyses on the FDS excluding red wine consumption.

As previously conducted for food groups in the UK Biobank [16], intraclass coefficients (ICCs) were calculated to test the reproducibility of the FDS over time and Spearman’s rank correlation coefficients were calculated to test the reliability of flavonoid intakes over time, using average flavonoid-rich food intakes from the Oxford WebQ dietary assessments at the second and third assessment (collected in February–April 2011 and June–August 2011) versus the fourth and fifth (October–December 2011 and April–June 2012). A subgroup of 21,543 participants who completed each of these dietary assessments were included in this analysis. Further, Spearman’s rank correlation coefficients were calculated to test the agreement between flavonoid intakes estimated from U.S Department of Agriculture (USDA) and Phenol-Explorer databases.

### Covariates

Sociodemographic, dietary, and lifestyle factors were self-reported at the baseline assessment between 2006 and 2010. Covariates considered in this study included: sex, age at recruitment, ethnicity, geographical region of recruitment, education, physical activity, alcohol intake, smoking status, energy intake, BMI, waist circumference, polypharmacy index, multimorbidity index, genetic risk of T2D (polygenic risk score (PRS) generated and issued by the UK Biobank for use upon request [17, 18]), Townsend deprivation index, family history of diabetes, hypercholesterolemia, hypertension, menopausal status, number of completed dietary assessments, wholegrain intake, sugar-sweetened beverage intake, red and processed meat intake, coffee intake and the healthful plant-based diet index [19]. Further details on how the covariates were classified for this study can be found in Table S1.

### Case ascertainment

Incident T2D cases were defined as primary type 2 diabetes mellitus according to the International Classification of Diseases 10th edition (ICD-10) (E11), using UK Biobank linked hospital inpatient data on admissions and diagnoses available until the 30th of September 2021 from the Hospital Episode Statistics (HES) for England, 31st of July 2021 for Scottish Morbidity Records (SMR), and 31st of March 2016 for the Patient Episode Database for Wales (PEDW). Follow-up for incident T2D analyses was censored at date of hospitalisation, death, or end of follow-up, whichever occurred first.

### Statistical analyses

Cox proportional hazards regression models were used to assess the relationship between the FDS, flavonoid subclass intakes, and intakes of individual flavonoid-rich foods, and incident T2D, producing hazard ratios (HR) and 95% confidence intervals (CI). The FDS was divided into sex-specific quartiles according to summed scores. Flavonoid intakes and major flavonoid-food contributors were also grouped into sex-specific quartiles, with the lowest quartile serving as the referent group across all analyses. The FDS, flavonoid subclass intakes, and flavonoid-rich foods were also modelled as continuous exposure variables when carrying out linear trend tests (P-trend).

Two models were used for adjustment in all analyses. Model 1 was adjusted for potential sociodemographic confounders; sex (female, male) and education (Low: CSEs or equivalent, O levels/GCSEs or equivalent; Medium: A levels/AS levels or equivalent, NVQ or HND or HNC or equivalent; High: College or University degree, other professional qualifications eg: nursing, teaching; unknown/missing/prefer not to say (6.6%)), stratified by age (<45 years, 45–, 50–, 55-, 60–, ≥65 years) at recruitment and geographical region of recruitment (ten UK regions). Model 2 was further adjusted for body mass index (BMI) (≤18·5 kg/m^2^, 18·5–24·9 kg/m^2^, 25·0–29·9 kg/m^2^, ≥30 kg/m^2^, or unknown/missing (0.1%)), waist circumference (continuous scale, cm), ethnicity (Asian, Black, Multiple, White, Other, or unknown/missing (0.3%)), physical activity (METs hr/week in quintiles, or unknown/missing (1.8%)), smoking status (never, previous, current, or unknown/missing (0.2%)), alcohol intake (<1 g/day, 1–7 g/day, 8–15 g/day, 16+ g/day, or unknown/missing (16.3%)), energy intake (continuous scale, kJ/day), polypharmacy index (total number of self-reported medications taken at baseline; 0, 1–3, 4–6, 7–9, >10, or unknown/missing (0.0%)), multimorbidity index (number of pre-existing long-term conditions; 0, 1, 2, or >3), hypercholesterolemia (no, yes), hypertension (no, yes), Townsend deprivation index (quintiles from low to high deprivation index, or unknown/missing (0.1%)), family history of diabetes (no, yes), menopausal status (no, yes, not sure (among women), or unknown/missing (0.1%)), PRS (tertiles from low to high PRS for T2D, or unknown/missing (2.1%)), number of completed dietary assessments (continuous scale, ranging between 2 and 5), wholegrain intake (continuous scale, servings/day), sugar-sweetened beverage intake (continuous scale, servings/day), red and processed meat intake (continuous scale, servings/day), and coffee intake (continuous scale, servings/day). Sensitivity analyses were also conducted, providing an alternative statistical model which involved replacing wholegrain, sugar-sweetened beverages, red and processed meat, and coffee intake with the healthful plant-based diet index (with a score ranging from 31 to 84 points) as a potential confounder. Further, a sensitivity analysis excluding study participants with less than two years of follow-up time to examine whether observed associations were due to reverse causality was also carried out. On completion of the Schoenfeld residuals test, there was no evidence to suggest the violation of the proportional hazards assumption.

To investigate effect modification, stratified analyses were carried out across potential risk modifiers smoking status (never, ever), sex (male, female), BMI (<25, ≥25 kg/m^2^), education (low: GSEs/O-Levels/GCSEs or equivalent, NVQ/HND/HNC/A-Levels/AS-Levels or equivalent; high: Other professional qualifications, College/university degree), ethnicity (white, non-white), and alcohol intake (<1 g/day, >1 g/day). Further, heterogeneity was assessed across strata of polygenic risk (T2D) (PRS tertiles; low, intermediate, high). Likelihood ratio tests (LRT) were used to test for potential effect modification of associations between the FDS and T2D risk by covariates. Multiplicative terms between the FDS (continuous) and covariates along with the main effect term were added to fully adjusted Cox regression models. LRTs were used to compare models with and without multiplicative terms.

A mediation analysis was also carried out to assess the association between potential mediators (biomarkers of obesity/sugar metabolism, inflammation, kidney and liver function, and lipid metabolism) on the FDS-T2D pathway. Details on the rationale for the choice of potential mediators and on the statistical methods are provided in the Supplementary Appendix.

Stata version 17.0 (Stata Corp LP, College Station, TX) was used to conduct all analyses. All reported *p* values were two-sided, with statistical significance set at *p* < 0.05 for main analyses. To account for multiple testing in flavonoid-rich food and flavonoid subclass analyses, Bonferroni correction was used [20].

## Results

### Characteristics of the study population

Baseline characteristics and key nutrient intakes among 113,097 study participants with data available from a minimum of 2 or more dietary assessments at baseline (mean (SD), 3.0 (0.9)) are presented across quartiles of the FDS in Table 1 and Table S2. During a median of 12 years of follow-up (1,358,384 person-years), 2628 cases of incident T2D cases occurred. Of the participants included, the median age (IQR) was 57 [12], 63,855 (56.5%) were female, 7864 (7.0%) were smokers, and 66,708 (59.0%) had a BMI over 25 kg/m^2^. The FDS ranged between 0 and 20.8 points, with higher scoring participants being more likely to be female, be of older age, be more physically active, and have a higher degree of education compared to participants with lower scores. The reproducibility ICC (range) of the FDS over time was 0.68 (0.1–30.5). For flavonoid intakes, Spearman’s coefficients for correlations over time ranged between 0.36 and 0.68 (Table S3). When comparing flavonoid intakes estimated through USDA and Phenol-Explorer databases, Spearman’s rank correlation coefficients were high for all comparisons (*r* > 0.79) except for the flavone subclass (Spearman’s *r* = 0.10) (Table S4).

**Table 1:** Baseline characteristics across quartiles (Q) of the Flavodiet Score in the UK Biobank (*n* = 113,097).

	Participants, No. (%)^a^				
Characteristics across FDS	Q1	Q2	Q3	Q4	Whole sample
Number of participants	28,497 (25.2)	29,979 (26.5)	29,241 (25.9)	25,380 (22.4)	113,097 (100.0)
T2D cases	807 (2.8)	704 (2.4)	603 (2.1)	514 (2.0)	2,628 (2.3)
FDS, mean (SD)	1.4 (0.7)	3.2 (0.5)	4.6 (0.4)	6.4 (1.0)	3.8 (1.9)
Sex-Female	16,133 (56.6)	15,619 (52.1)	18,839 (64.4)	13,264 (52.3)	63,855 (56.5)
Age at recruitment (years), mean (SD)	54.3 (8.1)	55.8 (7.9)	56.2 (7.7)	56.9 (7.4)	55.8 (7.8)
BMI (kg/m^2^), mean (SD)	27.0 (4.8)	26.5 (4.3)	26.3 (4.3)	26.3 (4.1)	26.5 (4.4)
Waist circumference (cm), mean (SD)	88.9 (13.4)	88.3 (12.7)	86.5 (12.5)	87.7 (12.5)	87.8 (12.8)
Energy intake (kJ/day), mean (SD)	8123 (1870)	8492 (1830)	8473 (1797)	8940 (1840)	8477 (1856)
Physical activity (MET-h/wk), mean (SD)	29.6 (38.4)	30.9 (37.7)	31.1 (38.6)	34.7 (40.7)	31.5 (38.8)
Ethnicity					
Asian	1730 (6.1)	1480 (4.9)	1137 (3.9)	955 (3.8)	5302 (4.7)
Black	113 (0.4)	124 (0.4)	92 (0.3)	62 (0.2)	391 (0.4)
Multiple	795 (2.8)	821 (2.7)	768 (2.6)	779 (3.1)	3163 (2.8)
White	25,542 (89.6)	27,282 (91.0)	27,031 (92.4)	23,362 (92.1)	103,218 (91.3)
Other^b^	222 (0.8)	179 (0.6)	127 (0.4)	127 (0.5)	655 (0.6)
Missing	95 (0.3)	92 (0.3)	86 (0.3)	95 (0.4)	368 (0.3)
Education					
Low	7739 (27.2)	7824 (26.1)	7687 (26.3)	5962 (23.5)	29,212 (25.8)
Medium	4843 (17.0)	4790 (16.0)	4510 (15.4)	3942 (15.5)	18,085 (16.0)
High	14,137 (49.6)	15,507 (51.7)	15,050 (51.5)	13,899 (54.8)	58,593 (51.8)
Missing	1178 (6.2)	1858 (6.2)	1994 (6.8)	1577 (6.2)	7207 (6.4)
Smoking status					
Never	16,608 (58.3)	17,396 (58.0)	17,357 (59.4)	14,218 (56.0)	65,579 (58.0)
Previous	9322 (32.7)	10,485 (35.0)	9970 (34.1)	9658 (38.1)	39,435 (34.9)
Current	2508 (8.8)	2035 (6.8)	1865 (6.4)	1456 (5.7)	7864 (7.0)
Missing	59 (0.2)	63 (0.2)	49 (0.2)	48 (0.2)	219 (0.2)
Drinking status					
Never	1023 (3.6)	747 (2.5)	809 (2.8)	536 (2.1)	3,115 (2.8)
Previous	1014 (3.6)	743 (2.5)	776 (2.7)	589 (2.3)	3,122 (2.8)
Current	26,441 (92.8)	28,462 (94.9)	27,643 (94.5)	24,240 (95.5)	106,786 (94.4)
Missing	19 (0.1)	27 (0.1)	13 (0.1)	15 (0.1)	74 (0.1)
Family history of diabetes	4784 (16.8)	4703 (15.7)	4591 (15.7)	3837 (15.1)	17,915 (15.8)
Hypertension	6264 (22.0)	6709 (22.4)	6290 (21.5)	6073 (23.9)	25,336 (22.4)
Hypercholesterolemia	3463 (12.2)	3807 (12.7)	3303 (11.3)	3406 (13.4)	13,979 (12.4)
Multimorbidity					
0 LTCs	11,417 (40.1)	12,259 (40.9)	11,542 (39.5)	9900 (39.0)	45,118 (39.9)
1 LTC	9481 (33.3)	9869 (32.9)	9589 (32.8)	8417 (33.2)	37,356 (33.0)
2 LTCs	4569 (16.0)	4913 (16.4)	4967 (17.0)	4414 (17.4)	18,863 (16.7)
≥3 LTCs	3030 (10.6)	2938 (9.8)	3143 (10.8)	2649 (10.4)	11,760 (10.4)
Polypharmacy					
0	9555 (33.5)	10,136 (33.8)	9514 (32.5)	8370 (33.0)	37,575 (33.2)
1–3	13,566 (47.6)	14,272 (47.6)	14,085 (48.2)	12,098 (47.7)	54,021 (47.8)
4–6	4090 (14.4)	4340 (14.5)	4375 (15.0)	3865 (15.2)	16,670 (14.7)
7–9	974 (3.4)	935 (3.1)	991 (3.4)	818 (3.2)	3718 (3.3)
≥10	312 (1.1)	294 (1.0)	276 (1.0)	227 (0.9)	1,109 (1.0)
Missing	0 (0.0)	2 (0.0)	0 (0.0)	2 (0.0)	4 (0.0)
Menopausal status					
Premenopausal	5238 (32.5)	4304 (27.6)	4917 (26.1)	3078 (23.2)	17,537 (27.5)
Postmenopausal	8450 (52.4)	9028 (57.8)	11,209 (59.5)	8301 (62.6)	36,988 (57.9)
Missing	18 (0.1)	23 (0.2)	15 (0.1)	16 (0.1)	72 (0.1)
PRS (T2D)					
Low	9097 (31.9)	9849 (32.9)	9606 (32.9)	8439 (33.3)	36,991 (32.7)
Medium	9339 (32.8)	9842 (32.8)	9532 (32.6)	8219 (32.4)	36,932 (32.7)
High	9433 (33.1)	9656 (32.2)	9521 (32.6)	8212 (32.4)	36,822 (32.6)
Missing	628 (2.2)	632 (2.1)	582 (2.0)	510 (2.0)	2352 (2.1)
Wholegrain intake, mean (SD)^c^	1.9 (1.4)	2.1 (1.4)	2.2 (1.4)	2.5 (1.5)	2.2 (1.5)
Sugar-sweetened beverage intake, mean (SD)^c^	0.7 (0.9)	0.5 (0.8)	0.4 (0.7)	0.4 (0.7)	0.5 (0.8)
Red and processed meat intake, mean (SD)^c^	0.9 (0.8)	0.9 (0.8)	0.9 (0.8)	0.9 (0.8)	0.9 (0.8)
Coffee intake, mean (SD)^c^	2.4 (1.6)	1.9 (1.4)	1.3 (1.2)	1.3 (1.2)	1.7 (1.5)

*Q* quartile, *FDS* flavodiet score, *BMI* body mass index, *MET* metabolic equivalent task, *PRS* polygenic risk score, *T2D* type 2 diabetes mellitus, *SD* standard deviation.

^a^Relative frequencies (%) include missing values which may not equate to 100%.

^b^Other includes any race or ethnic group not otherwise specified.

^c^Portion sizes were specified as a “serving” in the Oxford WebQ tool.

### Flavonoid intakes

Flavonoid intakes (total and subclass), major flavonoid-food contributors, and respective flavonoid compounds are shown in Table S5. Mean (SD) daily total flavonoid intake was 805.7 (±430.5) mg/d. The highest contributing subclass to total flavonoids was polymer intake (including proanthocyanidins), contributing 67%. Following this was flavan-3-ols, contributing 22% of total flavonoid intake. For both polymer and flavan-3-ol subclasses, intakes were primarily derived from tea consumption (70% for polymers and 80% for flavan-3-ols). The lowest contributing subclass to total flavonoids was flavones, with mean (SD) daily intakes of 1.1 (±0.9) mg/d, derived predominantly from peppers (17%) in this population.

### Flavodiet Score and incident type 2 diabetes risk

Minimal and multivariable-adjusted models for the associations between the FDS and T2D risk are shown in Tables 2 and S6. In the multivariable model adjusted for demographic and lifestyle characteristics (Model 2), a higher FDS (Quartile 4 (Q4)), representative of 6 servings of flavonoid-rich foods per day was associated with a 28% lower risk of incident T2D (HR: 0.72 [95%CI: 0.64–0.81], p_trend_ = <0.001), compared to a lower FDS Quartile 1 (Q1), representative of 1 serving of flavonoid-rich foods per day. Dose-response analysis indicated that a 1-point (serving/day) increment in FDS was associated with a 6% lower risk of T2D (HR: 0.94 [95%CI: 0.92–0.87], p_trend_ = <0.001). When removing red wine from the FDS, associations were only marginally attenuated, showing 6 servings of flavonoid-rich foods to be associated with a 26% lower risk of T2D (HR: 0.74 [95%CI: 0.66–0.84], p_trend_ = <0.001). Dose-response analysis also indicated that a 1-point increment (serving/day) in FDS (excluding red wine) was associated with a 5% lower risk of T2D (HR: 0.95 [95%CI: 0.93–0.97], p_trend_ = <0.001).

**Table 2:** Hazard ratios (95% confidence intervals) of type 2 diabetes across sex-specific quartiles (Q) of the Flavodiet Score (*n* = 113,097).

	Flavodiet Score quartiles					
	Q1	Q2	Q3	Q4	*P*-trend	1-point increments in Flavodiet score
FDS, mean (SD)	1.4 (0.7)	3.3 (0.5)	4.6 (0.4)	6.4 (1.0)	<0.001	
Cases/total	807/28,497	704/29,979	603/29,241	514/25,380		
Person-years	341,248	360,341	351,445	305,349		1,358,384
HR (95% CI)						
Model 1	1.00^a^	0.76 (0.68–0.84)	0.69 (0.62–0.76)	0.61 (0.55–0.68)	<0.001	0.91 (0.89–0.93)
Model 2	1.00^a^	0.86 (0.77–0.95)	0.77 (0.69–0.86)	0.72 (0.64–0.81)	<0.001	0.94 (0.92–0.97)

	Flavodiet Score quartiles (excluding red wine)					
	Q1	Q2	Q3	Q4	*P*-trend	1-point increments in Flavodiet score
FDS, mean (SD)	1.1 (0.6)	2.9 (0.4)	4.3 (0.4)	5.9 (1.0)	<0.001	
Cases/total	811/29,274	632/29,174	624/29,306	561/25,343		
Person-years	350,479	350,874	352,603	304,427		1,358,384
HR (95% CI)						
Model 1	1.00^a^	0.75 (0.67–0.83)	0.72 (0.65–0.80)	0.68 (0.61–0.76)	<0.001	0.93 (0.91–0.95)
Model 2	1.00^a^	0.83 (0.75–0.93)	0.79 (0.70–0.88)	0.74 (0.66–0.84)	<0.001	0.95 (0.93–0.97)

Model 1 was adjusted for sex and education; stratified by age (5-year categories) and region.

Model 2 was adjusted for sex, BMI, waist circumference, ethnicity, physical activity, smoking status, alcohol intake, education, energy intake, polypharmacy index, multimorbidity index, Townsend deprivation index, family history of diabetes, hypercholesterolemia, hypertension, menopausal status, PRS (T2D), number of completed dietary assessments, and intake of wholegrains, red and processed meat, sugar-sweetened beverages, and coffee; stratified by age (5-year categories) and region.

*Q* quartile, *FDS* flavodiet score, *BMI* body mass index, *PRS* polygenic risk score, *T2D* type 2 diabetes mellitus, *HR* hazard ratios, *CI* confidence intervals.

^a^Reference categories.

*P*-trend is for linear trend.

### Flavonoid-rich food intake and incident type 2 diabetes risks

We then investigated which of the main flavonoid-food contributors were associated with a lower incidence of T2D (Table 3). In the multivariable model adjusted for demographic and lifestyle characteristics comparing high (Q4) to low (Q1) intakes, consuming 4 servings/day of tea was associated with a 21% lower T2D risk (HR: 0.79 [95% CI: 0.70–0.90], *p*_trend_ = <0.001), 1 serving/day of berries was associated with a 15% lower T2D risk (HR: 0.85 [95% CI: 0.74–0.98], *p*_trend_ = 0.01), and 1 serving/day of apples was associated with a 12% lower risk of T2D (HR: 0.88 [0.79–0.98], *p*_trend_ = 0.03). After Bonferroni correction, statistically significant associations remained only for tea intake.

**Table 3:** Hazard ratios (95% confidence intervals) of type 2 diabetes across flavonoid-rich foods (*n* = 113,097).

	Flavonoid-rich food intake quartiles				
	Q1	Q2	Q3	Q4	*P* trend
Red wine intake					
Intake (servings/d)^a^	0 (0–0)	0.3 (0.1–0.4)	1.0 (0.6–1.0)	1.7 (1.1–6.0)	
Cases/total	1,874/71,422	263/16,080	264/13,972	227/11,623	
Person-years	856,382	193,745	168,617	139,639	
HR (95% CI)					
Model 1	1.00^b^	0.62 (0.54–0.70)	0.67 (0.59–0.76)	0.66 (0.57–0.75)	**<0.001**
Model 2	1.00^b^	0.81 (0.71–0.92)	0.84 (0.74–0.96)	0.75 (0.65–0.87)	**<0.001**
Tea intake					
Intake (servings/d)^a^	0 (0–0)	1.0 (0.2–1.8)	2.5 (2.0–3.4)	4.0 (3.5–4.0)	
Cases/total	559/21,440	629/25,996	720/33,414	720/32,247	
Person-years	256,771	312,590	401,947	387,076	
HR (95% CI)					
Model 1	1.00^b^	0.90 (0.80–1.01)	0.75 (0.67–0.84)	0.74 (0.66–0.83)	**<0.001**
Model 2	1.00^b^	1.03 (0.91–1.15)	0.89 (0.79–1.00)	0.79 (0.70–0.90)	**<0.001**
Berry intake					
Intake (servings/d)^a^	0 (0–0)	0.3 (0.1–0.3)	0.5 (0.3–0.5)	1.0 (0.6–4.0)	
Cases/total	1,874/72,473	244/12,871	281/15,212	229/12,541	
Person-years	868,926	154,658	183,350	151,451	
HR (95% CI)					
Model 1	1.00^b^	0.75 (0.66–0.86)	0.76 (0.67–0.86)	0.79 (0.69–0.91)	**<0.001**
Model 2	1.00^b^	0.88 (0.77–1.01)	0.85 (0.75–0.97)	0.85 (0.74–0.98)	0.01
Apple intake					
Intake (servings/d)^a^	0 (0–0)	0.3 (0.1–0.3)	0.5 (0.4–0.7)	1.0 (0.8–4.0)	
Cases/total	1,317/50,434	356/16,769	463/22,264	492/23,630	
Person-years	604,998	201,076	268,171	284,140	
HR (95% CI)					
Model 1	1.00^b^	0.83 (0.73–0.93)	0.80 (0.72–0.89)	0.76 (0.69-0.85)	**<0.001**
Model 2	1.00^b^	0.92 (0.81–1.03)	0.92 (0.82–1.02)	0.88 (0.79–0.98)	0.03
Grape intake					
Intake (servings/d)^a^	0 (0–0)	0.3 (0.1–0.3)	0.5 (0.3–0.5)	1.0 (0.6–4.0)	
Cases/total	1,779/75,024	276/13,460	323/13,667	250/10,946	
Person-years	901,022	161,758	164,249	131,346	
HR (95% CI)					
Model 1	1.00^b^	0.88 (0.77–1.00)	1.02 (0.91–1.15)	0.97 (0.85–1.11)	0.68
Model 2	1.00^b^	0.95 (0.83–1.08)	1.03 (0.91–1.16)	0.95 (0.83–1.09)	0.38
Orange intake					
Intake (servings/d)^a^	0 (0–0)	0.3 (0.1–0.3)	0.5 (0.4–0.8)	1.0 (0.8–5.5)	
Cases/total	1,511/62,341	287/14,104	417/19,591	413/17,061	
Person-years	748,089	169,577	235,462	205,256	
HR (95% CI)					
Model 1	1.00^b^	0.85 (0.75–0.97)	0.88 (0.79–0.99)	1.00 (0.90–1.12)	0.83
Model 2	1.00^b^	0.97 (0.85–1.10)	0.95 (0.85–1.06)	1.02 (0.91–1.14)	0.92
Grapefruit intake					
Intake (servings/d)^a^	0 (0–0)	0.2 (0.1–0.2)	0.3 (0.3–0.3)	0.5 (0.4–2.0)	
Cases/total	2,504/105,848	27/2,102	51/2,622	46/2,525	
Person-years	1,271,082	25,232	31,641	30,430	
HR (95% CI)					
Model 1	1.00^b^	0.55 (0.38–0.80)	0.80 (0.61–1.06)	0.72 (0.54–0.96)	0.01
Model 2	1.00^b^	0.72 (0.49–1.06)	0.91 (0.69–1.21)	0.81 (0.60–1.09)	0.11
Onion intake					
Intake (servings/d)^a^	0 (0–0)	0.1 (0.1–0.1)	0.3 (0.2–0.3)	0.5 (0.3–2.7)	
Cases/total	1,352/55,929	426/19,721	325/16,436	525/21,011	
Person-years	671,697	237,146	197,642	251,890	
HR (95% CI)					
Model 1	1.00^b^	0.95 (0.85–1.06)	0.86 (0.76–0.97)	1.08 (0.98–1.20)	0.07
Model 2	1.00^b^	0.99 (0.89–1.11)	0.91 (0.80–1.03)	1.02 (0.92–1.13)	0.73
Pepper intake					
Intake (servings/d)^a^	0 (0–0)	0.1 (0.1–0.1)	0.3 (0.2–0.3)	0.5 (0.3–3.0)	
Cases/total	1,846/72,794	321/16,921	221/11,757	240/11,625	
Person-years	872,969	203,708	141,721	139,987	
HR (95% CI)					
Model 1	1.00^b^	0.80 (0.71–0.90)	0.81 (0.71–0.94)	0.92 (0.80–1.05)	0.06
Model 2	1.00^b^	0.92 (0.81–1.04)	0.94 (0.81–1.08)	1.02 (0.89–1.17)	0.80
Dark chocolate intake					
Intake (servings/d)^a^	0 (0–0)	0.08 (0.05–0.08)	0.13 (0.1–0.2)	0.25 (0.2–2.3)	
Cases/total	2,334/96,761	87/5,470	93/5,374	114/5,486	
Person-years	1,116,503	66,003	64,811	65,997	
HR (95% CI)					
Model 1	1.00^b^	0.68 (0.55–0.84)	0.70 (0.57–0.87)	0.85 (0.70–1.02)	0.09
Model 2	1.00^b^	0.91 (0.73–1.13)	0.92 (0.75–1.14)	1.04 (0.86–1.26)	0.53

Model 1 was adjusted for sex and education; stratified by age (5-year categories) and region.

Model 2 was adjusted for sex, BMI, waist circumference, ethnicity, physical activity, smoking status, alcohol intake (excluding red wine analysis), education, energy intake, polypharmacy index, multimorbidity index, Townsend deprivation index, family history of diabetes, hypercholesterolemia, hypertension, menopausal status, PRS (T2D), number of completed dietary assessments, and intake of wholegrains, red and processed meat, sugar-sweetened beverages, and coffee; stratified by age (5-year categories) and region.

*Q* quartile, *BMI* body mass index, *T2D* type 2 diabetes mellitus, *PRS* polygenic risk score, *HR* hazard ratios, *CI* confidence intervals.

^a^Intake values are median (range) and portion sizes were specified as a “serving” in the Oxford WebQ tool.

^b^Reference categories.

*P* trend is for linear trend.

*P* trend in bold indicates *P* < 0.005, Bonferroni corrected.

### Flavonoid intake and incident type 2 diabetes risks

Associations between flavonoid subclass intakes and risk of T2D across quartiles are shown in Tables S7 and S8. Comparing high (Q4) to low (Q1) flavonoid subclass intakes, a higher intake of anthocyanins, flavan-3-ols, flavonols, flavones, polymers, and proanthocyanidins was associated with a 19%, 26%, 28%, 19%, 26%, and 27% lower risk of T2D after multivariable adjustments for demographic and lifestyle factors respectively. These associations remained statistically significant after Bonferroni correction. When assessing flavonoid subclass intake derived from Phenol-Explorer and T2D risk, results remained unchanged for all subclasses apart from flavones, where significant associations were lost upon multivariable adjustment (Table S9).

### Subgroup and sensitivity analyses

Stratified associations between the FDS and T2D are shown in Table S10, with no indication of heterogeneity across smoking status, sex, BMI, education, ethnicity or alcohol intake (*P*_interaction_ > 0.05). In the multivariable model adjusted for demographic and lifestyle characteristics, a higher FDS (Q4) compared to a lower FDS (Q1) across strata of genetic T2D risk only showed statistically significant inverse associations with incident T2D in intermediate and high-risk groups (HR: 0.71 [95%CI: 0.57–0.90], *p*_trend_ = 0.02; HR: 0.71 [95%CI: 0.60–0.84], *p*_trend_ = <0.001) (Table S11). However, there was no statistical heterogeneity in associations across strata of genetic risk. Further, when adjusting for the healthful plant-based diet index as a potential confounder, significant inverse associations remained for a higher FDS (Q4) compared to a lower FDS (Q1) (Table S12). Excluding study participants with ≤2 years of follow-up did not notably alter results (Table S13).

### Mediation analysis

Mediation analyses based on biomarker measurements were carried out to explore potential biological mechanisms (Table 4). Results showed BMI, IGF-1, C-reactive protein, cystatin C, urate, gamma glutamyl transferase (GGT) and alanine aminotransferase (ALT) to potentially mediate the FDS-T2D associations by 2–5%. Cumulative mediation analyses of significant mediators showed the observed associations between the FDS and T2D to be mediated by a proportion of 28% overall. Other factors (including creatinine, aspartate aminotransferase (AST), LDL-direct cholesterol or lipoprotein(a)) were not identified as potential mediators between the FDS and T2D risk.

**Table 4:** Mediation analysis between Flavodiet Score and type 2 diabetes.

	Flavodiet Score (1-point increments)							
	Participants, No.	Total effect (HR; 95% CI)^a^	*P* value	Direct effect (HR; 95% CI)^a^	*P* value	Natural indirect effect (HR; 95% CI)^a^	*P* value	Proportion mediated Log(NIE) / (Log(NIE)+log(NDE))
Potential mediators (HR; 95% CI)^b^								
Obesity and sugar metabolism								
BMI	80,877	0.957 [0.930–0.985]	0.003	0.960 [0.932–0.987]	0.005	0.998 [0.996–0.999]	0.001	5%
IGF-1	76,111	0.949 [0.921–0.977]	<0.001	0.950 [0.923–0.978]	0.001	0.999 [0.998–1.000]	0.001	2%
Inflammatory biomarkers								
C-reactive protein	76,377	0.948 [0.921–0.977]	<0.001	0.949 [0.921–0.978]	0.001	0.999 [0.998–0.999]	<0.001	2%
Kidney function								
Cystatin C	76,528	0.948 [0.921–0.977]	<0.001	0.950 [0.923–0.979]	0.001	0.998 [0.997–0.999]	<0.001	4%
Urate	76,453	0.948 [0.921–0.976]	<0.001	0.949 [0.922–0.977]	0.001	0.999 [0.998–1.000]	0.004	2%
Creatinine	76,480	0.948 [0.921–0.977]	<0.001	0.948 [0.921–0.976]	<0.001	1.000 [0.999–1.000]	0.579	NA
Liver function								
Gamma glutamyl transferase	76,484	0.951 [0.923–0.979]	0.001	0.951 [0.924–0.980]	0.001	0.999 [0.999–1.000]	0.001	2%
Alanine aminotransferase	76,506	0.951 [0.923–0.979]	0.001	0.953 [0.925–0.981]	0.001	0.998 [0.997–0.999]	<0.001	4%
Aspartate aminotransferase	76,262	0.950 [0.922–0.978]	0.001	0.950 [0.922–0.978]	0.001	1.000 [0.999–1.000]	0.937	NA
Lipid metabolism								
LDL-direct Cholesterol	76,394	0.949 [0.921–0.977]	<0.001	0.949 [0.922–0.977]	0.001	1.000 [0.999–1.000]	0.089	NA
Lipoprotein(a)	61,625	0.934 [0.903–0.965]	<0.001	0.934 [0.903–0.966]	<0.001	1.000 [0.999–1.000]	0.803	NA

*Q* quartile, *BMI* body mass index, *T2D* type 2 diabetes mellitus, *PRS* polygenic risk, *HR* hazard ratios, *CI* confidence intervals.

^a^Hazard Ratios with 95% Confidence Intervals (CI) for Flavodiet Score (1-point increments), adjusted for sex, BMI (excluding when considered as a potential mediator), waist circumference, ethnicity, physical activity, smoking status, alcohol intake, education, energy intake, polypharmacy index, multimorbidity index, Townsend deprivation index, family history of diabetes, hypercholesterolemia, hypertension, menopausal status, PRS (T2D), number of completed dietary assessments, and intake of wholegrains, red and processed meat, sugar-sweetened beverages, and coffee; stratified by age (5-year categories) and region.

^b^Potential mediators are modelled on the continuous scale.

## Discussion

For the first time in a population-based study, we examined the relationship between consumption of a flavonoid-rich diet as reflected by a higher FDS with risk of T2D. Results showed that a greater FDS, characterised by an average of 6 servings of flavonoid-rich foods per day (from several sources) was associated with a 26% lower risk of T2D. This association did not differ across groups of high genetic T2D risk or other covariates. Mediation analyses indicated that the protective association of flavonoid intake with T2D risk may be mediated, in part, by benefits of a flavonoid-rich diet on obesity and sugar metabolism (BMI and IGF-1), basal inflammation (C-reactive protein levels), kidney function (cystatin C and urate), and liver status (GGT and ALT). Of the major food contributors to flavonoid intake, red wine, tea (black and green), berry and apple intake were inversely associated with T2D risk.

While our study was the first to examine associations between the FDS, a novel tool that allows a real-world compatible operationalization of a flavonoid-rich diet, and T2D risk in a large prospective cohort, associations observed for flavonoid subclass intakes are in agreement with several previous studies. In a meta-analysis of 9 prospective cohort studies, higher dietary intakes of flavanols, flavonols, flavan-3-ols, and isoflavones were associated with up to a 14% lower risk of T2D compared to lower intakes [9]. Discrepancies in findings on flavanone intakes and T2D risk currently exist; an observational study in nondiabetic participants in the PREDIMED trial has previously shown inverse associations between flavanone intakes and T2D risk [21], while a large US prospective study showed positive associations [22]. In line with meta-analyses on flavonoid subclass intake and T2D risk [9, 23], our study found no associations between flavanone intakes and T2D. This lack of association may be attributed to fruit juice consumption, which largely contributes to flavanone intakes [24] (38% orange juice in this study), and has previously been associated with increasing the risk of T2D due to its high glycaemic load [25].

Tea, berries, apples, grapes, oranges, grapefruit, sweet peppers, onions, and dark chocolate were considered as major contributors to flavonoid intake and were therefore considered in the FDS. In a meta-analysis of 16 cohort studies, linear inverse associations were reported between tea consumption and T2D risk [26]. Likewise, meta-analyses on berry fruits, apples, grapes, and grapefruit and risk of T2D have demonstrated similar associations [27–29]. Similar to the meta-analysis carried out by Halvorsen et al. [29] on fruit and vegetable consumption and risk of T2D, we also reported no significant associations between orange and onion intake and T2D. As one of the few meta-analyses to have published on fruit and vegetable subtypes, Halvorsen et al. [29] did not report on associations between ‘other’ vegetable intake, including peppers. Of the previous studies published on fruit and vegetable intake and risk of T2D, only one study considered pepper intake within ‘other vegetables’, reporting inverse associations [30]. In contrast to results from our analyses where no significant associations were found between pepper intake and T2D risk, inverse associations reported by Villegas et al. [30] may partly be explained by peppers being grouped with ‘other’ vegetables, exerting a greater combined benefit. Therefore, direct comparisons between our findings cannot be made. Contrary to findings from a meta-analysis of randomised controlled trials reporting on the beneficial effects of cocoa and dark chocolate consumption on cardiovascular health (including T2D) [10], our results showed no significant associations between dark chocolate intake and risk of T2D, potentially due to the high number of participants with zero intakes (85.6%).

In this study we investigated the potential mechanisms by which flavonoids might impact T2D risk, using mediation analysis. In agreement with previous studies on flavonoid intakes and weight maintenance [31], our analysis suggests that lower T2D risk among those with a flavonoid-rich diet may be largely mediated by a lower BMI. However, in multivariable-adjusted models, associations were only marginally attenuated when BMI and waist circumference were included as potential confounders. In turn, our finding underlines that flavonoid intake may reduce weight gain through numerous mechanisms, including increased satiety [32] and energy metabolism [33]. Chronic inflammation has also been shown to play a major role in the development of metabolic dysfunction and particularly hyperglycaemia [34]. In line with our previous work where we showed that higher anthocyanin and flavonol intake was associated with anti-inflammatory effects [35], mediation analyses from this study also suggest that basal inflammation plays a considerable role in mediating the protective effects of a flavonoid-rich diet. These results are further supported by mechanistic insights from in vitro and animal studies suggesting higher flavonoid intakes to decrease T2D risk through reduced inflammation and oxidative stress [36, 37]. In addition to lower inflammation, better liver function may explain lower T2D among persons with higher FDS in our study. Again, these results are in agreement with previous studies to suggest that habitual flavonoid intake may protect against liver dysfunction [38], while non-alcoholic fatty liver disease is considered an independent diabetes risk factor [39]. Similarly, it has been proposed that flavonoids may enhance kidney function [40], and our findings suggest that habitual flavonoid intake may indeed protect against early kidney dysfunction before diabetes is diagnosed.

Several other mechanisms may explain the potential beneficial effects of a diet rich in flavonoids on T2D. Antidiabetic effects of several subclasses are suggested to arise through improved insulin signalling and secretion; firstly, through regulation of insulin-responsive glucose transporters e.g., GLUT4, whereby translocation facilitates glucose transportation and uptake in the body, and secondly, through the promotion of pancreatic β-cell proliferation, attenuating oxidative stress and apoptosis [37]. The anthocyanins, flavan-3-ols, and flavonols have raised particular interest due to their beneficial antidiabetic effects surrounding improved insulin resistance and glycemia metabolism [41]. The strong mediation by body fatness we observed may suggest that flavonoids exert beneficial effects in the early phase of diabetes development before manifesting hyperglycaemia.

In this study, a higher intake of red wine, tea, berries, and apples was associated with lower risks of T2D, even after adjusting for several demographic and lifestyle characteristics as potential confounders. This indicates that the observed beneficial effects on health may not be solely explained through overall improved diet quality, but rather something specifically present in flavonoid-rich plant foods. Due to its significant contribution to total anthocyanin intake (41% in this study), red wine was added as a FDS component. However, it is notable that on exclusion of red wine from the score, inverse associations were only marginally attenuated between a higher FDS and incidence of T2D.

Although the USDA database was used to estimate flavonoid intakes in this UK cohort, upon assessing the agreement between flavonoid intakes estimated from the USDA and Phenol-Explorer databases, all flavonoid subclasses showed excellent agreement (*r* > 0.79), except for the flavone subclass (Spearman’s *r* = 0.10). This is consistent with a previous study which compared flavonoid intakes derived from USDA and Phenol-explorer databases [42]. The association between flavone intake based on Phenol-Explorer data and diabetes risk was not statistically significant, possibly because flavone intake levels according to Phenol-Explorer were mostly driven by orange juice consumption, unlike USDA-derived flavone intakes [43].

The strengths of this study include the prospective design, large sample size, and long follow-up time of 12 years. Upon completion of subgroup analyses, this study showed no heterogeneity across strata of potential confounders. Sensitivity analysis excluding cases that occurred within the first 2 years of follow-up showed almost no change in results, supporting the assumption that associations are not a result of reverse causality. However, there are also some limitations to consider. Firstly, the UK Biobank cohort is made up of British middle-aged adults, >90% of which are of White-European ancestry which limits the generalisability of results to other non-European and ethnic groups. To better represent habitual dietary preferences, we used dietary data from participants who completed at least two 24-h dietary assessments, supported by our finding of a good reproducibility of the FDS over time based on these assessments. Although the Oxford WebQ dietary questionnaire has shown reasonable validity against objective biomarkers [44], we cannot rule out over- or underreporting and recall bias. Further, it can be assumed that in some cases, flavonoid sub-class intakes have been misclassified due to specific food items not being represented in the Oxford WebQ dietary questionnaire e.g., types of berries were not accounted for individually, but were grouped into one overarching category, ‘berries’. Additionally, flavonoid intake values may have also been misclassified through being unable to directly match an Oxford WebQ food item with a USDA flavonoid code, potentially attenuating risk estimates. Statistical mediation effects in our study were modest, with 28% of the association between the FDS and T2D attributable to mediators. This modest proportion may be due to potential additional, unmeasured mediators and regression dilution related to single biomarker measurements. It must also be considered that although multivariable-adjusted models accounted for several confounding factors, findings from this study were ascertained through observational data, possibly exposing associations to residual confounding.

## Conclusion

In summary, in this large prospective cohort of 113,097 middle-aged adults, a higher Flavodiet Score, achieved via a diet abundant in specific flavonoid-rich foods was associated with lower risks of incident T2D, irrespective of genetic predisposition and other established risk factors for T2D. Further, the observed inverse associations may be mediated by the beneficial effects of flavonoids on obesity/sugar metabolism, inflammation, and kidney and liver function. This study supports the current advice on increasing fruit consumption to reduce T2D risk, but points to a specific role for berries and apples. Encouraging an achievable increase in habitual intake of specific flavonoid-rich foods and beverages, namely tea, berries, and apples may lower T2D risk.

### Supplementary information

Supplementary Appendix

## Data Availability

UK Biobank data can be requested by all bona fide researchers for approved projects, including replication, through https://www.ukbiobank.ac.uk/. This research was conducted using UK Biobank funded and sourced data (application 64426).
