# Supplementary material for: Higher habitual intakes of flavonoids and flavonoid-rich foods are associated with a lower incidence of type 2 diabetes in the UK Biobank cohort
Source: Nutr Diabetes. 2024 May 22;14:32. doi: 10.1038/s41387-024-00288-0 (PMC11111454; doi:10.1038/s41387-024-00288-0)
Supplement: Supplementary file 1 — Supplementary Appendix [file 41387_2024_288_MOESM1_ESM.docx]

**Supplementary Appendix - Higher habitual intakes of flavonoids and flavonoid-rich foods are associated with a lower incidence of type 2 diabetes in the UK Biobank cohort**

Contents

[**Text S1.** Dietary assessment and flavonoid intakes 2](#_Toc165374272)

[**Text S2.** Mediation analysis 3](#_Toc165374273)

[**Table S1.** Covariate coding and categorisation information 4](#_Toc165374274)

[**Table S2.** Key nutrient intakes across quartiles (Q) of Flavodiet Score (N=113,097) 7](#_Toc165374275)

[**Table S3.** Spearman’s rank correlation coefficients for flavonoids intakes over time 8](#_Toc165374276)

[**Table S4**. Differences in flavonoid intakes estimated using USDA and Phenol-Explorer databases 9](#_Toc165374277)

[**Table S5.** Flavonoid intakes, major flavonoid-food contributors, and flavonoid subclass compounds 10](#_Toc165374278)

[**Table S6.** Hazard ratios (95% confidence intervals) of type 2 diabetes across quartiles (Q) of the Flavodiet Score, stratified by sex 11](#_Toc165374279)

[**Table S7.** Hazard ratios (95% confidence intervals) of type 2 diabetes across sex-specific quartiles (Q) of total-flavonoid and flavonoid-subclass intake (n=113,097) 12](#_Toc165374280)

[**Table S8.** Hazard ratios (95% confidence intervals) of type 2 diabetes across quartiles (Q) total-flavonoid and flavonoid-subclass intake, stratified by sex 13](#_Toc165374281)

[**Table S9.** Hazard ratios (95% confidence intervals) of type 2 diabetes across sex-specific quartiles (Q) of total-flavonoid and flavonoid-subclass intake derived from Phenol-Explorer (n=113,097) 15](#_Toc165374282)

[**Table S10.** Flavodiet Score and incident type 2 diabetes stratified by risk factors for diabetes 16](#_Toc165374283)

[**Table S11.** Multivariable adjusted hazard ratios (95% confidence intervals) of type 2 diabetes across strata of genetic type 2 diabetes risk, Flavodiet Score modelled as quartiles 17](#_Toc165374284)

[**Table S12.** Sensitivity analyses showing hazard ratios (95% confidence intervals) across sex-specific quartiles (Q) of the Flavodiet Score and type 2 diabetes, further adjusting for the healthful plant-based diet index as a potential confounder (n=113,097) 18](#_Toc165374285)

[**Table S13.** Sensitivity analyses showing hazard ratios (95% confidence intervals) across sex-specific quartiles (Q) of the Flavodiet Score and type 2 diabetes, restricting analyses to participant with at least two years of follow-up time (n=112,122) 19](#_Toc165374286)

[**Figure S1**. Flowchart of included participants 20](#_Toc165374287)

[**References** 21](#_Toc165374288)

## **Text S1.** Dietary assessment and flavonoid intakes

Dietary information was collected using the Oxford WebQ 24-hour dietary questionnaire, which asked about the frequency of consumption of >200 types of food and >30 beverages over the previous 24-hour period. Between 2009 and 2012, a large subset (n=210,950) of UK Biobank participants completed ≥1 Oxford WebQ, issued on five separate occasions (1). The Oxford WebQ has been tested for validity and reproducibility against objective nutritional biomarkers and compared with an interviewer-administered 24-hr recall to represent a better approximation of true dietary intake (2-4).

Given that major dietary sources of flavonoids tend to be very to be very similar across the globe (5-8), flavonoid content of foods and beverages from the Oxford WebQ was estimated using the US Department of Agriculture (USDA) database (9-11) – the largest existing flavonoid database and the most common method for estimating flavonoid intakes (12).The main data preparation steps to estimate flavonoid intake in the UK Biobank involved matching each Oxford WebQ food item with a USDA flavonoid code. All potential Oxford WebQ food-item-combinations and corresponding UK Nutrient Databank (UKNDB) (2013) food codes (13, 14) were made available in grams per day by Perez-Cornago et al. (15) to replace the older UK McCance and Widdowson’s “The Composition of Foods” 6^th^ edition (2002) food composition codes (16). For composite foods (recipes), flavonoid values for the specific ingredient were assigned. Flavonoid subclasses were calculated by summing the flavonoid compounds corresponding to a specific flavonoid subclass. Total flavonoid intakes were then calculated by summing the six main subclass groups (excluding the separate proanthocyanidins subclass). Final flavonoid intake values (mg/d) were calculated by multiplying the Oxford WebQ food intake (g/d) (using data from UK Biobank Returned Datasets Catalogue (ID: 4423)), with the flavonoid subclass content value (mg/g). Due to low consumption of isoflavone-rich foods in the UK population, isoflavone intakes were not estimated (17). Oxford WebQ food items that could not been matched with a USDA flavonoid code were assigned a value of zero.

## **Text S2.** Mediation analysis

The *paramed* package in Stata (18, 19) was used to conduct this analysis. Using two models, this parametric regression approach estimates the total, direct, and indirect effects of the treatment: indirect associations (natural indirect effect [NIE]) of the mediator conditional on the treatment and covariates, and direct associations (natural direct effect [NDE]) of the outcome conditional on the treatment, mediator, and covariates. The following potential mediators were selected based on previous studies: BMI (20) and IGF-1 (21) for obesity and sugar metabolism; C-reactive protein (22) for inflammation; cystatin C (23), urate (24), and creatinine (25) for kidney function; gamma glutamyl transferase (26), alanine aminotransferase (27), and aspartate aminotransferase (28) for liver function; and LDL-cholesterol (29) and lipoprotein(a) (30) for lipid metabolism. The percentage proportion of the association between the FDS and T2D mediated through one of the potential mediators of interest was calculated by dividing the log of the indirect effect HR by the sum of the log of the indirect effect HR and the log of the direct effect HR (log(NIE) / (log(NIE)+log(NDE)).

| **Table S1.** Covariate coding and categorisation information | | |
| --- | --- | --- |
| **Variables** | **Categorisation** | **UK Biobank variable description and data-field ID** |
| **Outcome** |  |  |
| Type 2 diabetes | No; Yes | ICD-10: Type 2 diabetes mellitus (E11) |
| **Demographics** |  |  |
| Age | 5-year categories (<45 years, 45–, 50–, 55-, 60–, ≥65 years) | Age at recruitment (ID: 21022)^†^ |
| Sex | Female; Male | Sex (ID: 31)^†^ |
| Ethnicity | Asian, Black, Multiple, White, Other; Unknown/Missing | Ethnic background (ID: 21000)^†^ |
| Region | London; Wales; North-West England; North-East England; Yorkshire; West Midlands; East Midlands; South-East England; South-West England; Scotland | UK Biobank assessment centre (ID:54)^†^ |
| **Socioeconomic status** |  |  |
| Education | Low: CSEs or equivalent, O levels/GCSEs or equivalent; Medium: A levels/AS levels or equivalent, NVQ or HND or HNC or equivalent; High: College or University degree, other professional qualifications eg: nursing, teaching; Unknown/Missing | Qualifications (ID: 6138)^†^ |
| Townsend deprivation index | Quintiles from least to most deprived; Unknown/ Missing | Townsend deprivation index (ID: 189)^†^ |
| **Diet and Lifestyle** |  |  |
| Alcohol intake | <1g/d; 1-7g/d; 8-15g/d; 16+g/d; Unknown/Missing | Alcohol intake frequency (ID:1558)^†^; Average weekly/monthly red wine intake (ID:1568/ID:4407)^†^; Average weekly/monthly white wine intake (ID:1578/ID:4418)^†^; Average weekly/monthly beer intake (ID: 1588/ID:4429)^†^; Average weekly/monthly spirits intake (ID: 4440/ ID:1598)^†^; Average weekly/monthly fortified wine intake (ID:1608/ ID:4451)^†^; Average weekly/monthly intake of other alcoholic drinks (ID: 5364/ ID:4462)^†^ |
| Smoking status | Never; Previous; Current; Unknown/Missing | Smoking status (ID: 20116)^†^ |
| Physical activity | METs hr/week quintiles; Unknown/Missing | Duration of walks (ID: 874)^†^; Number of days/week walked 10+ minutes (ID: 864)^†^; Duration of moderate activity (ID: 894)^†^; Number of days/week of moderate physical activity 10+ minutes (ID: 884)^†^; Duration of vigorous activity (ID: 914)^†^; Number of days/week of vigorous physical activity 10+ minutes (ID: 904)^†^ |
| Energy intake | Energy intake (kJ/day) (mean value across dietary assessments 1-5). | Energy (ID: 26002)^‡^ |
| Number of dietary assessments completed | Number of dietary assessments completed (ranging from 2-5) | Number of diet questionnaires completed (ID: 20077)^‡^ |
| Wholegrain intake | Number of servings reported yesterday | Diet by 24-hour recall (category ID: 100090)^‡^ |
| Sugar-sweetened beverage intake | Number of servings reported yesterday | Diet by 24-hour recall (category ID: 100090)^‡^ |
| Red and processed meat intake | Number of servings reported yesterday | Diet by 24-hour recall (category ID: 100090)^‡^ |
| Coffee intake | Number of servings reported yesterday | Diet by 24-hour recall (category ID: 100090)^‡^ |
| Healthful plant-based diet index | Quartiles from low to high adherence (ranging from 31-84) | Diet by 24-hour recall (category ID: 100090)^‡^ |
| **Health status** |  |  |
| BMI | Underweight (<18.5 kg/m^2^); Healthy weight (18.5-24.99 kg/m^2^); Overweight (25-29.99 kg/m^2^); Obese (≥30 kg/m^2^); Unknown/Missing | BMI (ID: 21001)^§^ |
| Waist circumference | Waist circumference (cm) | Waist circumference (ID: 48)^§^ |
| Menopausal status | No; Yes; Not sure (hysterectomy/other reason); Men; Unknown/Missing | Had menopause (women only) (ID: 2724)^†^ |
| Family history of diabetes | No; Yes | Illness of mother (ID: 20110)^†^ (Mother was diagnosed with diabetes); Illness of Father (ID: 20107)^†^ (Father was diagnosed with diabetes) |
| Hypercholesterolemia | No; Yes | Medication for cholesterol, blood pressure or diabetes (men) (ID: 6177)^†^; Medication for cholesterol, blood pressure, diabetes, or take exogenous hormones (women) (ID: 6153)^†^; Non-cancer illness diagnosed by nurse during verbal interview (ID: 20002)^¶^ |
| Hypertension | No; Yes | Vascular/heart problems diagnosed by doctor (heart attack, angina, stroke) (ID: 6150)^†^; Medication for cholesterol, blood pressure or diabetes (men) (ID: 6177)^†^; Medication for cholesterol, blood pressure, diabetes, or take exogenous hormones (women) (ID: 6153)^†^; Non-cancer illness diagnosed by nurse during verbal interview (ID: 20002)^¶^ |
| Multimorbidity | Number of pre-existing long-term conditions (0, 1, 2, ≥3) | Non-cancer illness diagnosed by nurse during verbal interview (ID: 20002)^¶^; Cancer diagnosed by doctor (ID: 2453)^†^ |
| Polypharmacy | Total number of self-reported medications taken at baseline (0, 1-3, 4-6, 7-9, ≥10; Unknown/Missing) | Number of treatments/medications taken (ID: 137)^¶^ |
| PRS (T2D) | Tertiles from low to high PRS for T2D; Unknown/Missing | Standard PRS for type 2 diabetes (T2D) (ID: 26285)^\|\|^ |
| **Blood biomarkers** |  |  |
| IGF-1 | IGF-1 (nmol/L) | IGF-1 (ID: 30770)^§^ |
| C-reactive protein | C-reactive protein (mg/L) | C-reactive protein (ID: 30710)^§^ |
| Cystatin C | Cystatin C (mg/L) | Cystatin C (ID: 30720)^§^ |
| Urate | Urate (umol/L) | Urate (ID: 30880)^§^ |
| Creatinine | Creatinine (umol/L) | Creatinine (ID: 30700)^§^ |
| Gamma glutamyltransferase | Gamma glutamyltransferase (U/L) | Gamma glutamyltransferase (ID: 30730)^§^ |
| Alanine aminotransferase | Alanine aminotransferase (U/L) | Alanine aminotransferase (ID: 30620)^§^ |
| Aspartate aminotransferase | Aspartate aminotransferase (U/L) | Aspartate aminotransferase (ID: 30650)^§^ |
| LDL cholesterol | LDL-direct (mmol/L) | LDL-direct (ID: 30780)^§^ |
| Lipoprotein(a) | Lipoprotein(a) (nmol/L) | Lipoprotein(a) (ID:30790)^§^ |
| ^†^Data collected at recruitment via touchscreen questionnaire (initial assessment visit (2006-2010)  ^‡^Data collected from 24-hr online Oxford WebQ dietary questionnaire (assessment centre (April 2009 to September 2010; on-line cycle 1 (February 2011 to April 2011); on-line cycle 2 (June 2011 to September 2011); on-line cycle 3 (October 2011 to December 2011); on-line cycle 4 (April 2012 to June 2012)  ^§^Physical measurements (initial assessment visit (2006-2010)  ^¶^Data collected via verbal interview (initial assessment visit (2006-2010)  ^\|\|^Genomics data (using blood samples from initial assessment visit (2006-2010)  *Abbreviations: BMI, body mass index; MET, metabolic equivalent task; CVD, cardiovascular disease; PRS, polygenic risk score; T2D, type 2 diabetes mellitus* | | |
|  |  |  |

## **Table S2.** Key nutrient intakes across quartiles (Q) of Flavodiet Score (N=113,097)

| **Key nutrient intakes** |  | **Mean (SD)** | |  |
| --- | --- | --- | --- | --- |
| **Flavodiet score quartiles** | **Q1** | **Q2** | **Q3** | **Q4** |
| Participants, No. (%) | 28,497 (25.2) | 29,979 (26.5) | 29,241 (22.9) | 25,380 (22.4) |
| Flavodiet score | 1.4 (0.7) | 3.2 (0.5) | 4.6 (0.4) | 6.4 (1.0) |
| Energy (kJ/d) | 8123.3 (1870.7) | 8424.1 (1830.1) | 8473.5 (1797.2) | 8940.4 (1840.7) |
| Alcohol (g/d) | 7.8 (9.5) | 8.9 (9.7) | 8.2 (9.4) | 10.8 (11.4) |
| Magnesium (mg/d) | 307.2 (78.0) | 327.8 (78.3) | 333.9 (77.5) | 370.1 (85.4) |
| Zinc (mg/d) | 9.2 (2.7) | 9.6 (2.7) | 9.8 (2.6) | 10.4 (2.8) |
| Cholesterol (mg/d) | 167.9 (96.0) | 172.2 (93.8) | 172.4 (92.3) | 175.6 (98.2) |
| Carbohydrate (g/d) | 240.6 (67.8) | 250.2 (66.8) | 253.4 (65.3) | 269.1 (69.5) |
| % EI | 49.5 (8.6) | 49.7 (8.4) | 50.0 (8.0) | 50.4 (8.6) |
| Glucose (g/d) | 23.6 (10.2) | 25.8 (10.7) | 26.1 (10.9) | 31.0 (12.6) |
| % EI | 4.6 (1.8) | 4.8 (1.9) | 4.9 (1.9) | 5.5 (2.1) |
| Protein (g/d) | 76.7 (20.8) | 79.7 (20.1) | 80.9 (19.4) | 85.2 (20.8) |
| % EI | 16.0 (3.5) | 16.0 (3.3) | 16.1 (3.1) | 16.1 (3.1) |
| Fibre (g/d) | 15.7 (5.2) | 17.3 (5.4) | 18.0 (5.4) | 20.8 (6.3) |
| % EI | 1.6 (0.5) | 1.7 (0.5) | 1.7 (0.5) | 1.9 (0.5) |
| Total fat (g/d) | 71.2 (24.2) | 73.1 (24.1) | 73.7 (23.9) | 75.5 (25.5) |
| % EI | 32.7 (7.0) | 32.4 (6.8) | 32.5 (6.6) | 31.5 (6.9) |
| Saturated fat (g/d) | 26.5 (10.3) | 27.1 (10.3) | 27.4 (10.2) | 27.7 (10.8) |
| % EI | 12.1 (3.3) | 12.0 (3.2) | 12.0 (3.2) | 11.5 (3.3) |
| Polyunsaturated fat (g/d) | 6.2 (2.4) | 6.4 (2.4) | 6.5 (2.4) | 6.8 (2.6) |
| % EI | 5.7 (1.7) | 5.7 (1.7) | 5.8 (1.6) | 5.7 (1.7) |
| *Abbreviations: SD, standard deviation; Q, quartile; EI, energy intake* | | | | |

| **Table S3.** Spearman’s rank correlation coefficients for flavonoids intakes over time | | | | |  |
| --- | --- | --- | --- | --- | --- |
|  | **24-hr dietary recall cycles** | | | **Spearman’s rank correlation**^†^ |  |
|  | **T2 +T3** | **T4 +T5** | **T2 + T3 *vs.* T4 + T5** | **T2 + T3 *vs.* T4 + T5** | |
|  | **Participants, No.** | | |  | |
| Total flavonoids | 98,644 | 101,767 | 87,322 | 0.65 | |
| Flavanones | 95,993 | 99,206 | 83,081 | 0.47 | |
| Anthocyanins | 96,841 | 99,992 | 84,446 | 0.38 | |
| Flavan-3-ols | 98,588 | 101,714 | 87,229 | 0.68 | |
| Flavonols | 98,642 | 101,761 | 87,315 | 0.57 | |
| Flavones | 97,391 | 100,588 | 85,310 | 0.36 | |
| Polymers | 98,512 | 101,622 | 87,097 | 0.68 | |
| Proanthocyanidins | 98,598 | 101,721 | 87,243 | 0.51 | |
| ^†^Spearman’s rank correlation coefficients are calculated from the means of cycles 2 and 3, correlated with the means of cycles 4 and 5. | | | | | |

## **Table S4**. Differences in flavonoid intakes estimated using USDA and Phenol-Explorer databases

|  | **Mean flavonoid intakes (mg/d)^†^** | |  |
| --- | --- | --- | --- |
|  | **USDA** | **Phenol-Explorer** | **Spearman’s rank correlation** |
| Total flavonoids | 805.7 ± 430.5 | 546.0 ± 294.3 | 0.97 |
| Flavanones | 23.6 ± 23.0 | 38.5 ± 44.4 | 0.91 |
| Anthocyanins | 28.1 ± 27.1 | 35.8 ± 34.8 | 0.95 |
| Flavan-3-ols | 183.7 ± 147.4 | 244.7 ± 168.6 | 0.93 |
| Flavonols | 32.5 ± 16.0 | 68.1 ± 37.5 | 0.88 |
| Flavones | 1.1 ± 0.9 | 7.7 ± 8.1 | 0.10 |
| Polymers^‡^ | 536.8 ± 313.4 | 143.7 ± 88.1 | 0.92 |
| Proanthocyanidins | 356.3 ± 191.8 | 337.2 ± 210.7 | 0.79 |
| ^†^Values are mean ± SD.  ^‡^Total polymers from USDA include the proanthocyanidins subclass. | | | |

## **Table S5.** Flavonoid intakes, major flavonoid-food contributors, and flavonoid subclass compounds

|  | Intake (mg/d)^†^ | Top three contributors (%) to class intake) | Flavonoid compounds |
| --- | --- | --- | --- |
| Total flavonoids | 805.7 ± 430.5 | Tea (67.2%); apples (5.8%); red wine (4.7%) |  |
| Polymers^‡^ | 536.8 ± 313.4 | Tea (69.9%); apples (7.5%); red wine (3.3%) | Including proanthocyanidins (excluding monomers), theaflavins and thearubigins |
| Proanthocyanidins | 356.3 ± 191.8 | Tea (45.9%); apples (12.4%); red wine (6.7%) | Dimers, trimers, 4–6 mers, 7–10 mers, polymers and monomers |
| Flavan-3-ols | 183.7 ± 147.4 | Tea (80.4%); red wine (3.6%); apples (2.2%) | Catechins, epicatachins |
| Anthocyanins | 28.1 ± 27.1 | Red wine (40.6%); berries (24.4%); grapes (15.0%) | Cyanidin, delphinidin, malvidin, pelargonidin, petunidin, peonidin |
| Flavonols | 32.5 ± 16.0 | Tea (55.4%); onions (7.5%); apples (5.5%) | Quercetin, kaempferol, myricetin and isohamnetin |
| Flavanones^§^ | 23.6 ± 23.0 | Oranges (37.6%); red wine (6.0%); grapefruit (3.7%) | Eriodictyol, hesperetin, naringenin |
| Flavones | 1.1 ± 0.9 | Peppers (17.0%); grapes (10.5%); red wine (9.3%) | Luteolin and apigenin |
| ^†^Values are mean ± SD  ^‡^Total polymers include proanthocyanidins  ^§^Top three contributors to flavanone intakes excluding fruit juices. | | | |

| **Table S6.** Hazard ratios (95% confidence intervals) of type 2 diabetes across quartiles (Q) of the Flavodiet Score, stratified by sex | | | | | | | |
| --- | --- | --- | --- | --- | --- | --- | --- |
|  | | **Flavodiet Score quartiles** | | | | |  |
|  | | Q1 | Q2 | Q3 | Q4 | P-trend | P-interaction |
|  | |  | **Men** | | |  |  |
| FDS, mean (SD) | | 1.3 (0.7) | 3.3 (0.5) | 4.6 (0.4) | 6.3 (1.1) |  | 0.98 |
| Cases/total | | 452/12,364 | 454/14,360 | 317/10,402 | 337/12,116 |  |  |
| HR (95% CI)^†^ | | 1.00^‡^ | 0.87 (0.76-0.99) | 0.79 (0.68-0.93) | 0.74 (0.64-0.87) | 0.001 |  |
|  | |  | **Women** | | |  |  |
| FDS, mean (SD) | | 1.4 (0.7) | 3.2 (0.4) | 4.6 (0.4) | 6.5 (1.0) |  |  |
| Cases/total | | 355/16,133 | 250/15,619 | 286/18,839 | 177/13,264 |  |  |
| HR (95% CI)^†^ | | 1.00^‡^ | 0.84 (0.71-0.99) | 0.75 (0.63-0.88) | 0.68 (0.56-0.83) | <0.001 |  |
|  | | **Flavodiet Score quartiles (excluding red wine)** | | | | |  |
|  | | Q1 | Q2 | Q3 | Q4 | P-trend | P-interaction |
|  |  | **Men** | | |  |  |  |
| FDS, mean (SD) | 1.1 (0.6) | 2.9 (0.4) | 4.1 (0.3) | 5.7 (1.0) |  | 0.88 |  |
| Cases/total | 474/13,309 | 363/12,383 | 352/11,342 | 371/12,208 |  |  |  |
| HR (95% CI)^†^ | 1.00^‡^ | 0.83 (0.72-0.96) | 0.81 (0.70-0.94) | 0.77 (0.66-0.90) | 0.004 |  |  |
|  |  | **Women** | | |  |  |  |
| FDS, mean (SD) | 1.2 (0.6) | 2.9 (0.5) | 4.4 (0.4) | 6.1 (0.9) |  |  |  |
| Cases/total | 337/15,965 | 269/16,791 | 272/17,964 | 190/13,135 |  |  |  |
| HR (95% CI)^†^ | 1.00^‡^ | 0.83 (0.70-0.98) | 0.75 (0.63-0.89) | 0.70 (0.58-0.85) | <0.001 |  |  |
| ^†^Hazard Ratios with 95% Confidence Intervals (CI), adjusted for BMI, waist circumference, ethnicity, physical activity, smoking status, alcohol intake, education, energy intake, polypharmacy index, multimorbidity index, Townsend deprivation index, family history of diabetes, hypercholesterolemia, hypertension, menopausal status, PRS (T2D), number of completed dietary assessments, and intake of wholegrains, red and processed meat, sugar-sweetened beverages, and coffee; stratified by age (5-year categories) and region.  ^‡^Reference categories  P-trend is for linear trend.  Heterogeneity was tested by comparing two models – one without an interaction term between sex and Flavodiet Score (categorical), with a model that included an interaction term. The likelihood ratio test was used to produce P-interaction values.  *Abbreviations: Q, quartile; FDS, flavodiet score; SD, standard deviation; BMI, body mass index; PRS, polygenic risk; T2D, type 2 diabetes mellitus; HR, hazard ratios; CI, confidence intervals* | | | | | | | |

| **Table S7.** Hazard ratios (95% confidence intervals) of type 2 diabetes across sex-specific quartiles (Q) of total-flavonoid and flavonoid-subclass intake (n=113,097) | | | | | | |
| --- | --- | --- | --- | --- | --- | --- |
|  | | **Flavonoid intake quartiles** | | | | |
|  | Q1 | | Q2 | Q3 | Q4 | P-trend |
| **Total Flavonoids** |  | |  |  |  |  |
| Intake (mg/d)^†^ | 271.0 (0-463.0) | | 632.9 (444.5-799.4) | 946.8 (788.1-1125.6) | 1339.0 (1114.1-3611.4) |  |
| Cases/total | 794/28,276 | | 626/28,273 | 608/28,274 | 600/28,274 |  |
| HR (95% CI) |  | |  |  |  |  |
| Model 1 | 1.00^‡^ | | 0.75 (0.68-0.83) | 0.69 (0.62-0.77) | 0.66 (0.59-0.74) | **<0.001** |
| Model 2 | 1.00^‡^ | | 0.83 (0.75-0.93) | 0.80 (0.72-0.90) | 0.71 (0.63-0.81) | **<0.001** |
| **Flavanones** |  | |  |  |  |  |
| Intake (mg/d)^†^ | 1.6 (0-6.0) | | 10.7 (4.9-19.5) | 25.6 (16.3-38.0) | 50.0 (33.0-376.7) |  |
| Cases/total | 741/28,270 | | 648/28,275 | 627/28,273 | 612/28,279 |  |
| HR (95% CI) |  | |  |  |  |  |
| Model 1 | 1.00^‡^ | | 0.89 (0.80-0.99) | 0.85 (0.77-0.95) | 0.83 (0.74-0.92) | 0.04 |
| Model 2 | 1.00^‡^ | | 1.03 (0.92-1.14) | 1.02 (0.91-1.13) | 1.00 (0.90-1.12) | 0.85 |
| **Anthocyanins** |  | |  |  |  |  |
| Intake (mg/d)^†^ | 3.2 (0-8.8) | | 13.4 (5.5-22.0) | 29.0 (18.4-40.8) | 58.4 (39.7-307.2) |  |
| Cases/total | 833/28,273 | | 662/28,276 | 587/28,271 | 546/28,277 |  |
| HR (95% CI) |  | |  |  |  |  |
| Model 1 | 1.00^‡^ | | 0.76 (0.68-0.84) | 0.66 (0.59-0.73) | 0.60 (0.54-0.67) | **<0.001** |
| Model 2 | 1.00^‡^ | | 0.88 (0.79-0.97) | 0.86 (0.77-0.96) | 0.81 (0.72-0.91) | **<0.001** |
| **Flavan-3-ols** |  | |  |  |  |  |
| Intake (mg/d)^†^ | 39.2 (0-85.7) | | 128.3 (85.1-167.5) | 203.5 (164.4-248.6) | 306.9 (244.9-1903.7) |  |
| Cases/total | 781/28,276 | | 622/28,273 | 626/28,273 | 599/28,275 |  |
| HR (95% CI) |  | |  |  |  |  |
| Model 1 | 1.00^‡^ | | 0.76 (0.68-0.84) | 0.72 (0.65-0.80) | 0.68 (0.62-0.76) | **<0.001** |
| Model 2 | 1.00^‡^ | | 0.85 (0.76-0.94) | 0.80 (0.71-0.90) | 0.74 (0.65-0.83) | **<0.001** |
| **Flavonols** |  | |  |  |  |  |
| Intake (mg/d)^†^ | 13.4 (0-20.6) | | 26.3 (19.9-31.9) | 37.3 (31.6-43.9) | 51.4 (43.3-140.5) |  |
| Cases/total | 782/28,276 | | 628/28,272 | 620/28,273 | 598/28,276 |  |
| HR (95% CI) |  | |  |  |  |  |
| Model 1 | 1.00^‡^ | | 0.77 (0.69-0.85) | 0.71 (0.64-0.79) | 0.67 (0.60-0.74) | **<0.001** |
| Model 2 | 1.00^‡^ | | 0.87 (0.78-0.96) | 0.81 (0.73-0.91) | 0.72 (0.64-0.82) | **<0.001** |
| **Flavones** |  | |  |  |  |  |
| Intake (mg/d)^†^ | 0.3 (0-0.5) | | 0.7 (0.4-0.9) | 1.1 (0.8-1.6) | 2.0 (1.3-10.6) |  |
| Cases/total | 813/28,274 | | 679/28,269 | 578/28,275 | 558/28,279 |  |
| HR (95% CI) |  | |  |  |  |  |
| Model 1 | 1.00^‡^ | | 0.81 (0.73-0.90) | 0.69 (0.62-0.77) | 0.66 (0.59-0.73) | **<0.001** |
| Model 2 | 1.00^‡^ | | 0.96 (0.87-1.07) | 0.86 (0.77-0.96) | 0.81 (0.73-0.91) | **0.002** |
| **Polymers** |  | |  |  |  |  |
| Intake (mg/d)^†^ | 153.4 (0-280.9) | | 397.9 (259.0-528.4) | 637.3 (512.7-774.2) | 933.3 (761.4-2029.7) |  |
| Cases/total | 783/28,276 | | 612/28,275 | 616/28,273 | 617/28,273 |  |
| HR (95% CI) |  | |  |  |  |  |
| Model 1 | 1.00^‡^ | | 0.73 (0.66-0.82) | 0.69 (0.62-0.77) | 0.67 (0.60-0.74) | **<0.001** |
| Model 2 | 1.00^‡^ | | 0.85 (0.76-0.95) | 0.83 (0.74-0.93) | 0.74 (0.65-0.83) | **<0.001** |
| **Proanthocyanidins** |  | |  |  |  |  |
| Intake (mg/d)^†^ | 162.5 (0-331.1) | | 285.7 (229.6-337.3) | 387.5 (335.7-449.2) | 536.9 (445.8-2338.3) |  |
| Cases/total | 835/28,277 | | 645/28,273 | 591/28,273 | 557/28,274 |  |
| HR (95% CI) |  | |  |  |  |  |
| Model 1 | 1.00^‡^ | | 0.71 (0.64-0.79) | 0.62 (0.56-0.69) | 0.58 (0.52-0.65) | **<0.001** |
| Model 2 | 1.00^‡^ | | 0.83 (0.75-0.93) | 0.75 (0.67-0.85) | 0.73 (0.65-0.83) | **<0.001** |
| Model 1 was adjusted for sex and education; stratified by age (5-year categories) and region.  Model 2 was adjusted for sex, BMI, waist circumference, ethnicity, physical activity, smoking status, alcohol intake, education, energy intake, polypharmacy index, multimorbidity index, Townsend deprivation index, family history of diabetes, hypercholesterolemia, hypertension, menopausal status, PRS (T2D), number of completed dietary assessments, and intake of wholegrains, red and processed meat, sugar-sweetened beverages, and coffee; stratified by age (5-year categories) and region.  ^†^Intake values are median (range).  ^‡^Reference categories.  P-trend is for linear trend.  P-trend in bold indicates P-value <0.006, Bonferroni corrected.  *Abbreviations: Q, quartile; BMI, body mass index; PRS, polygenic risk score; T2D, type 2 diabetes mellitus; HR, hazard ratios; CI, confidence intervals.* | | | | | | |

| **Table S8.** Hazard ratios (95% confidence intervals) of type 2 diabetes across quartiles (Q) total-flavonoid and flavonoid-subclass intake, stratified by sex | | | | | | |
| --- | --- | --- | --- | --- | --- | --- |
| **Flavonoid intake quartiles** | | | | | | |
|  | Q1 | Q2 | Q3 | Q4 | P-trend | P-interaction |
|  |  | **Men** | |  |  |  |
| **Total Flavonoids** |  |  | |  |  |  |
| Cases/total | 453/12,312 | 359/12,309 | 384/12,310 | 364/12,311 |  | 0.30 |
| HR (95% CI)^†^ | 1.00^‡^ | 0.80 (0.69-0.92) | 0.84 (0.72-0.98) | 0.72 (0.62-0.85) | **<0.001** |  |
|  |  | **Women** | |  |  |  |
| Cases/total | 341/15,964 | 267/15,964 | 224/15,964 | 236/15,963 |  |  |
| HR (95% CI)^†^ | 1.00^‡^ | 0.89 (0.75-1.05) | 0.75 (0.62-0.90) | 0.70 (0.58-0.84) | **<0.001** |  |
|  |  | **Men** | |  |  |  |
| **Flavanones** |  |  | |  |  | 0.66 |
| Cases/total | 424/12,309 | 400/12,312 | 375/12,309 | 361/12,312 |  |  |
| HR (95% CI)^†^ | 1.00^‡^ | 1.08 (0.94-1.24) | 1.03 (0.90-1.19) | 1.01 (0.87-1.16) | 0.72 |  |
|  |  | **Women** | |  |  |  |
| Cases/total | 317/15,961 | 248/15,963 | 252/15,964 | 251/15,967 |  |  |
| HR (95% CI)^†^ | 1.00^‡^ | 0.96 (0.81-1.14) | 1.01 (0.85-1.20) | 1.00 (0.85-1.19) | 0.33 |  |
|  |  | **Men** | |  |  |  |
| **Anthocyanins** |  |  | |  |  | 0.42 |
| Cases/total | 492/12,311 | 380/12,310 | 349/12,308 | 339/12,313 |  |  |
| HR (95% CI)^†^ | 1.00^‡^ | 0.84 (0.73-0.96) | 0.86 (0.75-0.99) | 0.83 (0.72-0.97) | 0.03 |  |
|  |  | **Women** | |  |  |  |
| Cases/total | 341/15,962 | 282/15,966 | 238/15,963 | 207/15,964 |  |  |
| HR (95% CI)^†^ | 1.00^‡^ | 0.94 (0.80-1.10) | 0.87 (0.73-1.03) | 0.80 (0.66-0.96) | 0.01 |  |
|  |  | **Men** | |  |  |  |
| **Flavan-3-ols** |  |  | |  |  | 0.62 |
| Cases/total | 439/12,312 | 373/12,309 | 392/12,310 | 356/12,311 |  |  |
| HR (95% CI)^†^ | 1.00^‡^ | 0.87 (0.75-1.00) | 0.84 (0.72-0.98) | 0.73 (0.62-0.86) | **<0.001** |  |
|  |  | **Women** | |  |  |  |
| Cases/total | 342/15,964 | 249/15,964 | 234/15,963 | 243/15,964 |  |  |
| HR (95% CI)^†^ | 1.00^‡^ | 0.83 (0.70-0.98) | 0.75 (0.62-0.90) | 0.75 (0.62-0.91) | **0.005** |  |
|  |  | **Men** | |  |  |  |
| **Flavonols** |  |  | |  |  | 0.43 |
| Cases/total | 434/12,312 | 392/12,309 | 369/12,310 | 356/12,311 |  |  |
| HR (95% CI)^†^ | 1.00^‡^ | 0.93 (0.81-1.08) | 0.82 (0.71-0.96) | 0.75 (0.64-0.88) | **<0.001** |  |
|  |  | **Women** | |  |  |  |
| Cases/total | 348/15,964 | 236/15,963 | 251/15,963 | 233/15,965 |  |  |
| HR (95% CI)^†^ | 1.00^‡^ | 0.78 (0.66-0.93) | 0.81 (0.68-0.96) | 0.69 (0.57-0.83) | **<0.001** |  |
|  |  | **Men** | |  |  |  |
| **Flavones** |  |  | |  |  | 0.48 |
| Cases/total | 479/12,310 | 389/12,308 | 358/12,311 | 334/12,313 |  |  |
| HR (95% CI)^†^ | 1.00^‡^ | 0.91 (0.80-1.05) | 0.87 (0.76-1.01) | 0.81 (0.70-0.94) | 0.02 |  |
|  |  | **Women** | |  |  |  |
| Cases/total | 334/15,964 | 290/15,961 | 220/15,964 | 224/15,966 |  |  |
| HR (95% CI)^†^ | 1.00^‡^ | 1.03 (0.88-1.21) | 0.84 (0.70-1.00) | 0.82 (0.69-0.98) | 0.06 |  |
|  |  | **Men** | |  |  |  |
| **Polymers** |  |  | |  |  | 0.54 |
| Cases/total | 450/12,312 | 351/12,310 | 377/12,309 | 382/12,311 |  |  |
| HR (95% CI)^†^ | 1.00^‡^ | 0.83 (0.72-0.95) | 0.84 (0.73-0.98) | 0.77 (0.66-0.90) | **0.005** |  |
|  |  | **Women** | |  |  |  |
| Cases/total | 333/15,964 | 261/15,965 | 239/15,964 | 235/15,962 |  |  |
| HR (95% CI)^†^ | 1.00^‡^ | 0.89 (0.76-1.06) | 0.82 (0.68-0.98) | 0.69 (0.57-0.83) | **<0.001** |  |
|  |  | **Men** | |  |  |  |
| **Proanthocyanidins** |  |  | |  |  | 0.32 |
| Cases/total | 461/12,314 | 403/12,308 | 366/12,310 | 330/12,310 |  |  |
| HR (95% CI)^†^ | 1.00^‡^ | 0.91 (0.80-1.05) | 0.82 (0.70-0.95) | 0.77 (0.65-0.90) | **<0.001** |  |
|  |  | **Women** | |  |  |  |
| Cases/total | 374/15,963 | 242/15,965 | 225/15,963 | 227/15,964 |  |  |
| HR (95% CI)^†^ | 1.00^‡^ | 0.73 (0.62-0.87) | 0.68 (0.57-0.81) | 0.69 (0.67-0.83) | **<0.001** |  |
| ^†^Hazard Ratios with 95% Confidence Intervals (CI), adjusted for BMI, waist circumference, ethnicity, physical activity, smoking status, alcohol intake, education, energy intake, polypharmacy index, multimorbidity index, Townsend deprivation index, family history of diabetes, hypercholesterolemia, hypertension, menopausal status, PRS (T2D), number of completed dietary assessments, and intake of wholegrains, red and processed meat, sugar-sweetened beverages, and coffee; stratified by age (5-year categories) and region.  ^‡^Reference categories  P-trend is for linear trend.  P-trend in bold indicates P-value <0.006, Bonferroni corrected.  Heterogeneity was tested by comparing two models – one without an interaction term between sex and flavonoid subclass (categorical), with a model that included an interaction term. The likelihood ratio test was used to produce P-interaction values.  *Abbreviations: Q, quartile; BMI, body mass index; PRS, polygenic risk; T2D, type 2 diabetes mellitus; HR, hazard ratios; CI, confidence intervals* | | | | | |  |

| **Table S9.** Hazard ratios (95% confidence intervals) of type 2 diabetes across sex-specific quartiles (Q) of total-flavonoid and flavonoid-subclass intake derived from Phenol-Explorer (n=113,097) | | | | | | |
| --- | --- | --- | --- | --- | --- | --- |
|  | | **Flavonoid intake quartiles** | | | | |
|  | Q1 | | Q2 | Q3 | Q4 | P-trend |
| **Total Flavonoids** |  | |  |  |  |  |
| Intake (mg/d)^†^ | 179.4 (0-326.8) | | 430.0 (294.7-551.5) | 643.4 (526.9-775.0) | 909.2 (747.8-2143.9) |  |
| Cases/total | 788/28,275 | | 644/28,274 | 605/28,275 | 591/28,273 |  |
| HR (95% CI) |  | |  |  |  |  |
| Model 1 | 1.00^‡^ | | 0.78 (0.70-0.86) | 0.69 (0.62-0.77) | 0.66 (0.59-0.73) | **<0.001** |
| Model 2 | 1.00^‡^ | | 0.89 (0.80-0.99) | 0.81 (0.72-0.90) | 0.74 (0.65-0.83) | **<0.001** |
| **Flavanones** |  | |  |  |  |  |
| Intake (mg/d)^†^ | 1.1 (0-3.2) | | 7.3 (2.3-28.3) | 40.1 (20.2-68.8) | 95.1 (53.8-775.5) |  |
| Cases/total | 720/28,275 | | 644/28,274 | 668/28,285 | 596/28,263 |  |
| HR (95% CI) |  | |  |  |  |  |
| Model 1 | 1.00^‡^ | | 0.90 (0.81-1.00) | 0.93 (0.83-1.03) | 0.85 (0.76-0.94) | 0.09 |
| Model 2 | 1.00^‡^ | | 0.94 (0.85-1.05) | 1.09 (0.98-1.22) | 1.00 (0.89-1.11) | 0.49 |
| **Anthocyanins** |  | |  |  |  |  |
| Intake (mg/d)^†^ | 1.5 (0-11.7) | | 18.0 (5.3-29.3) | 38.9 (23.6-54.1) | 75.7 (50.7-516.8) |  |
| Cases/total | 812/28,275 | | 643/28,274 | 583/28,275 | 590/28,273 |  |
| HR (95% CI) |  | |  |  |  |  |
| Model 1 | 1.00^‡^ | | 0.76 (0.69-0.84) | 0.68 (0.61-0.75) | 0.67 (0.60-0.74) | **<0.001** |
| Model 2 | 1.00^‡^ | | 0.88 (0.79-0.98) | 0.87 (0.78-0.97) | 0.86 (0.77-0.97) | **0.002** |
| **Flavan-3-ols** |  | |  |  |  |  |
| Intake (mg/d)^†^ | 28.2 (0-103.5) | | 173.4 (84.2-240.5) | 299.6 (237.3-371.5) | 456.8 (367.4-1295.5) |  |
| Cases/total | 751/28,275 | | 644/28,274 | 619/28,275 | 614/28,273 |  |
| HR (95% CI) |  | |  |  |  |  |
| Model 1 | 1.00^‡^ | | 0.82 (0.74-0.91) | 0.75 (0.67-0.83) | 0.71 (0.64-0.80) | **<0.001** |
| Model 2 | 1.00^‡^ | | 0.93 (0.83-1.03) | 0.84 (0.75-0.95) | 0.75 (0.66-0.85) | **<0.001** |
| **Flavonols** |  | |  |  |  |  |
| Intake (mg/d)^†^ | 23.7 (0-40.1) | | 53.3 (38.6-66.0) | 78.7 (65.6-93.6) | 112.0 (93.0-584.1) |  |
| Cases/total | 773/28,275 | | 637/28,274 | 606/28,275 | 612/28,273 |  |
| HR (95% CI) |  | |  |  |  |  |
| Model 1 | 1.00^‡^ | | 0.78 (0.71-0.87) | 0.71 (0.64-0.79) | 0.70 (0.63-0.78) | **<0.001** |
| Model 2 | 1.00^‡^ | | 0.89 (0.80-0.99) | 0.83 (0.74-0.92) | 0.78 (0.69-0.88) | **<0.001** |
| **Flavones** |  | |  |  |  |  |
| Intake (mg/d)^†^ | 0.6 (0-1.7) | | 3.0 (1.4-6.1) | 7.9 (4.5-13.0) | 16.6 (10.4-123.3) |  |
| Cases/total | 755/28,275 | | 649/28,274 | 648/28,275 | 576/28,273 |  |
| HR (95% CI) |  | |  |  |  |  |
| Model 1 | 1.00^‡^ | | 0.84 (0.76-0.94) | 0.85 (0.77-0.95) | 0.76 (0.68-0.85) | **<0.001** |
| Model 2 | 1.00^‡^ | | 0.93 (0.84-1.04) | 1.00 (0.89-1.11) | 0.92 (0.82-1.03) | 0.50 |
| **Polymers** |  | |  |  |  |  |
| Intake (mg/d)^†^ | 33.9 (0-79.4) | | 108.1 (66.9-146.1) | 171.0 (134.8-213.8) | 251.5 (200.7-662.2) |  |
| Cases/total | 785/28,275 | | 638/28,274 | 620/28,275 | 585/28,273 |  |
| HR (95% CI) |  | |  |  |  |  |
| Model 1 | 1.00^‡^ | | 0.75 (0.68-0.84) | 0.68 (0.62-0.76) | 0.63 (0.57-0.70) | **<0.001** |
| Model 2 | 1.00^‡^ | | 0.90 (0.81-1.00) | 0.83 (0.74-0.93) | 0.75 (0.66-0.84) | **<0.001** |
| **Proanthocyanidins** |  | |  |  |  |  |
| Intake (mg/d)^†^ | 67.6 (0-173.5) | | 252.1 (147.1-338.0) | 407.2 (323.6-500.6) | 600.9 (485.8-1515.4) |  |
| Cases/total | 776/28,275 | | 631/28,274 | 630/28,275 | 591/28,273 |  |
| HR (95% CI) |  | |  |  |  |  |
| Model 1 | 1.00^‡^ | | 0.76 (0.68-0.84) | 0.71 (0.64-0.79) | 0.65 (0.58-0.72) | **<0.001** |
| Model 2 | 1.00^‡^ | | 0.89 (0.80-0.99) | 0.85 (0.75-0.95) | 0.73 (0.64-0.82) | **<0.001** |
| Model 1 was adjusted for sex and education; stratified by age (5-year categories) and region.  Model 2 was adjusted for sex, BMI, waist circumference, ethnicity, physical activity, smoking status, alcohol intake, education, energy intake, polypharmacy index, multimorbidity index, Townsend deprivation index, family history of diabetes, hypercholesterolemia, hypertension, menopausal status, PRS (T2D), number of completed dietary assessments, and intake of wholegrains, red and processed meat, sugar-sweetened beverages, and coffee; stratified by age (5-year categories) and region.  ^†^Intake values are median (range).  ^‡^Reference categories.  P-trend is for linear trend.  P-trend in bold indicates P-value <0.006, Bonferroni corrected.  *Abbreviations: Q, quartile; BMI, body mass index; PRS, polygenic risk score; T2D, type 2 diabetes mellitus; HR, hazard ratios; CI, confidence intervals.* | | | | | | |

| **Table S10.** Flavodiet Score and incident type 2 diabetes stratified by risk factors for diabetes | | | | | | | | | | | | |
| --- | --- | --- | --- | --- | --- | --- | --- | --- | --- | --- | --- | --- |
|  | | **Flavodiet score quartiles** | | | | | |  | | |  | |
|  | **Cases/**  **Total** | **Q1** | **Q2** | | **Q3** | | **Q4** | | P-trend | 1-point increments in Flavodiet score | | P- interaction |
| **Smoking status** |  |  | |  | |  |  | |  |  | | 0.59 |
| HR (95% CI)^†^ |  |  | |  | |  |  | |  |  | |  |
| Never | 1,203/  65,579 | 1.00^‡^ | | 0.83 (0.70-0.96) | | 0.72 (0.61-0.84) | 0.66 (0.55-0.79) | | <0.001 | 0.93 (0.90-0.96) | |  |
| Ever | 1,421/  47,299 | 1.00^‡^ | | 0.89 (0.77-1.03) | | 0.83 (0.71-0.98) | 0.76 (0.64-0.89) | | 0.004 | 0.97 (0.94-1.00) | |  |
| **Sex** |  |  | |  | |  |  | |  |  | | 0.98 |
| HR (95% CI)^†^ |  |  | |  | |  |  | |  |  | |  |
| Male | 1,560/  49,242 | 1.00^‡^ | | 0.87 (0.76-0.99) | | 0.79 (0.68-0.93) | 0.74 (0.64-0.87) | | 0.001 | 0.95 (0.92-0.98) | |  |
| Female | 1,068/63,855 | 1.00^‡^ | | 0.84 (0.71-0.99) | | 0.75 (0.63-0.88) | 0.68 (0.56-0.83) | | <0.001 | 0.94 (0.90-0.97) | |  |
| **BMI** |  |  | |  | |  |  | |  |  | | 0.65 |
| HR (95% CI)^†^ |  |  | |  | |  |  | |  |  | |  |
| <25.0 kg/m^2^ | 295/  46,275 | 1.00^‡^ | | 0.89 (0.64-1.23) | | 0.99 (0.70-1.39) | 0.94 (0.65-1.36) | | 0.61 | 0.98 (0.92-1.05) | |  |
| ≥ 25.0 kg/m^2^ | 2,323/  66,709 | 1.00^‡^ | | 0.86 (0.77-0.96) | | 0.75 (0.67-0.85) | 0.70 (0.62-0.80) | | <0.001 | 0.94 (0.92-0.96) | |  |
| **Education** |  |  | |  | |  |  | |  |  | | 0.26 |
| HR (95% CI)^†^ |  |  | |  | |  |  | |  |  | |  |
| Low | 1,106/  47,297 | 1.00^‡^ | | 0.82 (0.70-0.96) | | 0.74 (0.62-0.89) | 0.75 (0.62-0.91) | | <0.001 | 0.94 (0.91-0.98) | |  |
| High | 1,154/  58,593 | 1.00^‡^ | | 0.89 (0.76-1.04) | | 0.85 (0.71-1.00) | 0.71 (0.59-0.85) | | <0.001 | 0.95 (0.92-0.98) | |  |
| **Ethnicity** |  |  | |  | |  |  | |  |  | | 0.46 |
| HR (95% CI)^†^ |  |  | |  | |  |  | |  |  | |  |
| White | 2,391/103,218 | 1.00^‡^ | | 0.84 (0.75-0.94) | | 0.77 (0.68-0.86) | 0.72 (0.63-0.82) | | <0.001 | 0.95 (0.93-0.97) | |  |
| Non-White | 224/9,511 | 1.00^‡^ | | 1.02 (0.72-1.44) | | 0.76 (0.50-1.15) | 0.66 (0.42-1.03) | | 0.02 | 0.91 (0.84-0.99) | |  |
| **Alcohol intake** |  |  | |  | |  |  | |  |  | | 0.78 |
| HR (95% CI)^†^ |  |  | |  | |  |  | |  |  | |  |
| <1g/day | 197/5,472 | 1.00^‡^ | | 0.99 (0.68-1.45) | | 0.62 (0.41-0.94) | 0.60 (0.37-0.99) | | 0.06 | 0.92 (0.85-1.00) | |  |
| ≥1g/day | 1,781/89,225 | 1.00^‡^ | | 0.82 (0.72-0.93) | | 0.76 (0.66-0.88) | 0.68 (0.59-0.79) | | <0.001 | 0.94 (0.91-0.96) | |  |
| ^†^Hazard Ratios with 95% Confidence Intervals (CI), adjusted for sex (excluding subgroup analysis), BMI (excluding subgroup analysis), waist circumference, ethnicity (excluding subgroup analysis), physical activity, smoking status, alcohol intake (excluding subgroup analysis), education (excluding subgroup analysis), energy intake, polypharmacy index, multimorbidity index, Townsend deprivation index, family history of diabetes, hypercholesterolemia, hypertension, menopausal status, PRS (T2D), number of completed dietary assessments, and intake of wholegrains, red and processed meat, sugar-sweetened beverages, and coffee; stratified by age (5-year categories) and region.  ^‡^Reference categories.  P-trend is for linear trend.  Heterogeneity was tested by comparing two models – one without an interaction term between subgroup of interest and Flavodiet Score (categorical), with a model that included an interaction term. The likelihood ratio test was used to produce P-interaction values.  *Abbreviations: Q, quartile; BMI, body mass index; PRS, polygenic risk; T2D, type 2 diabetes mellitus; HR, hazard ratios; CI, confidence intervals.* | | | | | | | | | | | | |

| **Table S11.** Multivariable adjusted hazard ratios (95% confidence intervals) of type 2 diabetes across strata of genetic type 2 diabetes risk, Flavodiet Score modelled as quartiles | | | | | |  |
| --- | --- | --- | --- | --- | --- | --- |
| **Flavodiet score** | Q1 | Q2 | Q3 | Q4 | P-trend |  |
| **PRS – low** |  |  |  |  |  |  |
| Cases/total | 110/9,097 | 107/9,849 | 94/9,606 | 74/8,439 |  |  |
| HR (95% CI)^†^ | 1.00^‡^ | 1.00 (0.76-1.32) | 0.96 (0.72-1.30) | 0.79 (0.57- 1.09) | 0.36 |  |
| **PRS – Intermediate** |  |  |  |  |  |  |
| Cases/total | 227/9,339 | 210/9,842 | 173/9,532 | 141/8,219 |  |  |
| HR (95% CI)^†^ | 1.00^‡^ | 0.94 (0.78-1.15) | 0.82 (0.66-1.02) | 0.71 (0.57-0.90) | 0.02 |  |
| **PRS - High** |  |  |  |  |  |  |
| Cases/total | 454/9,433 | 371/9,656 | 323/9,521 | 294/8,212 |  |  |
| HR (95% CI)^†^ | 1.00^‡^ | 0.78 (0.68-0.90) | 0.70 (0.60-0.82) | 0.71 (0.60-0.84) | <0.001 |  |
| ^†^Hazard Ratios with 95% Confidence Intervals (CI), adjusted for sex, BMI, waist circumference, ethnicity, physical activity, smoking status, alcohol intake, education, energy intake, polypharmacy index, multimorbidity index, Townsend deprivation index, family history of diabetes, hypercholesterolemia, hypertension, menopausal status, number of completed dietary assessments, and intake of wholegrains, red and processed meat, sugar-sweetened beverages, and coffee; stratified by age (5-year categories) and region.  ^†^Reference categories.  P-trend is for linear trend.  *Abbreviations: Q, quartile; BMI, body mass index; PRS, polygenic risk; HR, hazard ratios; CI, confidence intervals.* | | | | | |  |

| **Table S12.** Sensitivity analyses showing hazard ratios (95% confidence intervals) across sex-specific quartiles (Q) of the Flavodiet Score and type 2 diabetes, further adjusting for the healthful plant-based diet index as a potential confounder (n=113,097) | | | | | | |
| --- | --- | --- | --- | --- | --- | --- |
|  | **Flavodiet Score quartiles** | | | | |  |
|  | Q1 | Q2 | Q3 | Q4 | P-trend | 1-point increments in Flavodiet score |
| FDS, mean (SD) | 1.4 (0.7) | 3.3 (0.5) | 4.6 (0.4) | 6.4 (1.0) | <0.001 |  |
| Cases/total | 807/28,497 | 704/29,979 | 603/29,241 | 514/25,380 |  |  |
| HR (95% CI)^†^ | 1.00^‡^ | 0.91 (0.82-1.01) | 0.87 (0.78-0.97) | 0.83 (0.74-0.94) | 0.008 | 0.97 (0.95-0.99) |
|  | **Flavodiet Score quartiles (excluding red wine)** | | | | |  |
|  | Q1 | Q2 | Q3 | Q4 | P-trend | 1-point increments in Flavodiet score |
| FDS, mean (SD) | 1.1 (0.6) | 2.9 (0.4) | 4.3 (0.4) | 5.9 (1.0) | <0.001 |  |
| Cases/total | 811/29,274 | 632/29,174 | 624/29,306 | 561/25,343 |  |  |
| HR (95% CI)^†^ | 1.00^‡^ | 0.88 (0.79-0.98) | 0.88 (0.79-0.98) | 0.87 (0.78-0.98) | 0.05 | 0.98 (0.96-1.00) |
| ^†^Hazard Ratios with 95% Confidence Intervals (CI), adjusted for sex, BMI, waist circumference, ethnicity, physical activity, smoking status, alcohol intake, education, energy intake, polypharmacy index, multimorbidity index, Townsend deprivation index, family history of diabetes, hypercholesterolemia, hypertension, menopausal status, PRS (T2D), number of completed dietary assessments and the healthful plant-based diet index; stratified by age (5-year categories) and region.  ^‡^Reference categories.  P-trend is for linear trend.  *Abbreviations: Q, quartile; FDS, flavodiet Score; BMI, body mass index; PRS, polygenic risk score; T2D, type 2 diabetes mellitus; HR, hazard ratios; CI, confidence intervals.* | | | | | | |

| **Table S13.** Sensitivity analyses showing hazard ratios (95% confidence intervals) across sex-specific quartiles (Q) of the Flavodiet Score and type 2 diabetes, restricting analyses to participant with at least two years of follow-up time (n=112,122) | | | | | | |
| --- | --- | --- | --- | --- | --- | --- |
|  | **Flavodiet score quartiles** | | | |  |  |
|  | Q1 | Q2 | Q3 | Q4 | P-trend | 1-point increments in Flavodiet score |
| FDS, mean (SD) | 1.4 (0.7) | 3.3 (0.5) | 4.6 (0.4) | 6.4 (1.0) | <0.001 |  |
| Cases/total | 709/28,222 | 620/29,716 | 520/28,989 | 457/25,195 |  |  |
| HR (95% CI) |  |  |  |  |  |  |
| Model 1 | 1.00^†^ | 0.76 (0.68-0.85) | 0.68 (0.60-0.76) | 0.62 (0.55-0.70) | <0.001 | 0.91 (0.90-0.93) |
| Model 2 | 1.00^†^ | 0.85 (0.76-0.95) | 0.74 (0.66-0.84) | 0.71 (0.62-0.81) | <0.001 | 0.94 (0.92-0.96) |
|  | **Flavodiet score quartiles (excluding red wine)** | | | |  |  |
|  | Q1 | Q2 | Q3 | Q4 | P-trend |  |
| FDS, mean (SD) | 1.1 (0.6) | 2.9 (0.4) | 4.3 (0.4) | 5.9 (1.0) | <0.001 |  |
| Cases/total | 710/28,987 | 554/28,924 | 540/29,052 | 502/25,159 |  |  |
| HR (95% CI) |  |  |  |  |  |  |
| Model 1 | 1.00^†^ | 0.75 (0.67-0.84) | 0.72 (0.64-0.80) | 0.70 (0.63-0.79) | <0.001 | 0.93 (0.91-0.96) |
| Model 2 | 1.00^†^ | 0.83 (0.74-0.92) | 0.76 (0.68-0.86) | 0.74 (0.66-0.84) | <0.001 | 0.95 (0.92-0.97) |
| Model 1 was adjusted for sex and education; stratified by age (5-year categories) and region.  Model 2 was adjusted for sex, BMI, waist circumference, ethnicity, physical activity, smoking status, alcohol intake, education, energy intake, polypharmacy index, multimorbidity index, Townsend deprivation index, family history of diabetes, hypercholesterolemia, hypertension, menopausal status, PRS (T2D), number of completed dietary assessments, and intake of wholegrains, red and processed meat, sugar-sweetened beverages, and coffee; stratified by age (5-year categories) and region.  ^†^Reference categories.  P-trend is for linear trend.  *Abbreviations: Q, quartile; FDS, flavodiet score; BMI, body mass index; PRS, polygenic risk score; T2D, type 2 diabetes mellitus; HR, hazard ratios; CI, confidence intervals.* | | | | | | |

## **Figure S1**. Flowchart of included participants

## **References**

1. Bradbury KE, Young HJ, Guo W, Key TJ. Dietary assessment in UK Biobank: an evaluation of the performance of the touchscreen dietary questionnaire. J Nutr Sci. 2018;7:e6-e.

2. Greenwood DC, Hardie LJ, Frost GS, Alwan NA, Bradbury KE, Carter M, et al. Validation of the Oxford WebQ Online 24-Hour Dietary Questionnaire Using Biomarkers. Am J Epidemiol. 2019;188(10):1858-67.

3. Galante J, Adamska L, Young A, Young H, Littlejohns TJ, Gallacher J, et al. The acceptability of repeat Internet-based hybrid diet assessment of previous 24-h dietary intake: administration of the Oxford WebQ in UK Biobank. British Journal of Nutrition. 2016;115(4):681-6.

4. Liu B, Young H, Crowe FL, Benson VS, Spencer EA, Key TJ, et al. Development and evaluation of the Oxford WebQ, a low-cost, web-based method for assessment of previous 24 h dietary intakes in large-scale prospective studies. Public Health Nutr. 2011;14(11):1998-2005.

5. Vogiatzoglou A, Mulligan AA, Lentjes MA, Luben RN, Spencer JP, Schroeter H, et al. Flavonoid intake in European adults (18 to 64 years). PloS one. 2015;10(5):e0128132.

6. Zamora-Ros R, Knaze V, Rothwell JA, Hémon B, Moskal A, Overvad K, et al. Dietary polyphenol intake in Europe: the European Prospective Investigation into Cancer and Nutrition (EPIC) study. European journal of nutrition. 2016;55:1359-75.

7. Bondonno NP, Lewis JR, Blekkenhorst LC, Bondonno CP, Shin JH, Croft KD, et al. Association of flavonoids and flavonoid-rich foods with all-cause mortality: The Blue Mountains Eye Study. Clinical Nutrition. 2020;39(1):141-50.

8. Bertoia ML, Rimm EB, Mukamal KJ, Hu FB, Willett WC, Cassidy A. Dietary flavonoid intake and weight maintenance: three prospective cohorts of 124 086 US men and women followed for up to 24 years. bmj. 2016;352.

9. US Department of Agriculture. USDA Database for the Flavonoid Content of Selected Foods, Release 3.2. Washington DC. 2015.

10. US Department of Agriculture. USDA's Expanded Flavonoid Database for the Assessment of Dietary Intakes, Release 1.1. Washington DC. 2015.

11. US Department of Agriculture. USDA Database for the Proanthocyanidin Content of Selected Foods, Release 2. Washington DC. 2015.

12. Haytowitz DB, Wu X, Bhagwat S. USDA database for the flavonoid content of selected foods, release 3.3. US Department of Agriculture. 2018;173.

13. Swan G, Dodhia S, Farron‐Wilson M, Powell N, Bush M. Food composition data and public health. Nutrition Bulletin. 2015;40(3):223-6.

14. Cambridge Uo. National Diet and Nutrition Survey Years 1-11, 2008-2019. In: Unit ME, editor. 19th Edition ed: NatCen Social Research; 2021.

15. Perez-Cornago A, Pollard Z, Young H, van Uden M, Andrews C, Piernas C, et al. Description of the updated nutrition calculation of the Oxford WebQ questionnaire and comparison with the previous version among 207,144 participants in UK Biobank. European Journal of Nutrition. 2021;60(7):4019-30.

16. McCance R, Widdowson E. The Composition of Foods, 6th summary edn. Compiled by the Food Standards Agency and Institute of Food Research: UK. 2002.

17. Mulligan A, Welch A, McTaggart A, Bhaniani A, Bingham S. Intakes and sources of soya foods and isoflavones in a UK population cohort study (EPIC-Norfolk). European journal of clinical nutrition. 2007;61(2):248-54.

18. Emsley RA, Liu H, Dunn G, Valeri L, VanderWeele TJ. Paramed: A command to perform causal mediation analysis using parametric models. The Stata Journal. 2014.

19. Dunn G, Emsley R, Liu H, Landau S, Green J, White I, et al. Evaluation and validation of social and psychological markers in randomised trials of complex interventions in mental health: a methodological research programme. Health Technology Assessment (Winchester, England). 2015;19(93):1-116.

20. Vazquez G, Duval S, Jacobs Jr DR, Silventoinen K. Comparison of body mass index, waist circumference, and waist/hip ratio in predicting incident diabetes: a meta-analysis. Epidemiologic reviews. 2007;29(1):115-28.

21. Teppala S, Shankar A. Association between serum IGF-1 and diabetes among US adults. Diabetes care. 2010;33(10):2257-9.

22. Wang X, Bao W, Liu J, OuYang Y-Y, Wang D, Rong S, et al. Inflammatory markers and risk of type 2 diabetes: a systematic review and meta-analysis. Diabetes care. 2013;36(1):166-75.

23. Sahakyan K, Lee K, Shankar A, Klein R. Serum cystatin C and the incidence of type 2 diabetes mellitus. Diabetologia. 2011;54:1335-40.

24. Bhole V, Choi JWJ, Kim SW, De Vera M, Choi H. Serum uric acid levels and the risk of type 2 diabetes: a prospective study. The American journal of medicine. 2010;123(10):957-61.

25. Bao X, Gu Y, Zhang Q, Liu L, Meng G, Wu H, et al. Low serum creatinine predicts risk for type 2 diabetes. Diabetes/Metabolism Research and Reviews. 2018;34(6):e3011.

26. Kunutsor SK, Abbasi A, Adler AI. Gamma-glutamyl transferase and risk of type II diabetes: an updated systematic review and dose-response meta-analysis. Annals of epidemiology. 2014;24(11):809-16.

27. Sattar N, Scherbakova O, Ford I, O’Reilly DSJ, Stanley A, Forrest E, et al. Elevated Alanine Aminotransferase Predicts New-Onset Type 2 Diabetes Independently of Classical Risk Factors, Metabolic Syndrome, and C-Reactive Protein in the West of Scotland Coronary Prevention Study. Diabetes. 2004;53(11):2855-60.

28. Hanley AJG, Williams K, Festa A, Wagenknecht LE, D’Agostino RB, Jr., Kempf J, et al. Elevations in Markers of Liver Injury and Risk of Type 2 Diabetes: The Insulin Resistance Atherosclerosis Study. Diabetes. 2004;53(10):2623-32.

29. Seo MH, Bae JC, Park SE, Rhee EJ, Park CY, Oh KW, et al. Association of lipid and lipoprotein profiles with future development of type 2 diabetes in nondiabetic Korean subjects: a 4-year retrospective, longitudinal study. The Journal of Clinical Endocrinology & Metabolism. 2011;96(12):E2050-E4.

30. Mora S, Kamstrup PR, Rifai N, Nordestgaard BG, Buring JE, Ridker PM. Lipoprotein (a) and risk of type 2 diabetes. Clinical chemistry. 2010;56(8):1252-60.
